# Spectral density-based and measure-preserving ABC for partially observed diffusion processes. An illustration on Hamiltonian SDEs

**DOI:** 10.1007/s11222-019-09909-6

**Published:** 2019-11-05

**Authors:** Evelyn Buckwar, Massimiliano Tamborrino, Irene Tubikanec

**Affiliations:** Institute for Stochastics, Johannes Kepler University Linz, Altenberger Straße 69, 4040 Linz, Austria

**Keywords:** Approximate Bayesian computation, Likelihood-free inference, Stochastic differential equations, Numerical splitting schemes, Invariant measure, Neural mass models

## Abstract

Approximate Bayesian computation (ABC) has become one of the major tools of likelihood-free statistical inference in complex mathematical models. Simultaneously, stochastic differential equations (SDEs) have developed to an established tool for modelling time-dependent, real-world phenomena with underlying random effects. When applying ABC to stochastic models, two major difficulties arise: First, the derivation of effective summary statistics and proper distances is particularly challenging, since simulations from the stochastic process under the same parameter configuration result in different trajectories. Second, exact simulation schemes to generate trajectories from the stochastic model are rarely available, requiring the derivation of suitable numerical methods for the synthetic data generation. To obtain summaries that are less sensitive to the intrinsic stochasticity of the model, we propose to build up the statistical method (e.g. the choice of the summary statistics) on the underlying structural properties of the model. Here, we focus on the existence of an invariant measure and we map the data to their estimated invariant density and invariant spectral density. Then, to ensure that these model properties are kept in the synthetic data generation, we adopt measure-preserving numerical splitting schemes. The derived property-based and measure-preserving ABC method is illustrated on the broad class of partially observed Hamiltonian type SDEs, both with simulated data and with real electroencephalography data. The derived summaries are particularly robust to the model simulation, and this fact, combined with the proposed reliable numerical scheme, yields accurate ABC inference. In contrast, the inference returned using standard numerical methods (Euler–Maruyama discretisation) fails. The proposed ingredients can be incorporated into any type of ABC algorithm and directly applied to all SDEs that are characterised by an invariant distribution and for which a measure-preserving numerical method can be derived.

**Electronic supplementary material:**

The online version of this article (10.1007/s11222-019-09909-6) contains supplementary material, which is available to authorized users.

## Introduction

Over the last decades, stochastic differential equations (SDEs) have become an established and powerful tool for modelling time-dependent, real-world phenomena with underlying random effects. They have been successfully applied to a variety of scientific fields, ranging from biology over finance to physics, chemistry, neuroscience and others. Diffusion processes obtained as solutions of SDEs are typically characterised by some underlying structural properties whose investigation and preservation are crucial. Examples are boundary properties, symmetries or the preservation of invariants or qualitative behaviour such as the ergodicity or the conservation of energy. Here, we focus on a specific structural property, namely the existence of a unique invariant measure. Besides the modelling, it is of primary interest to estimate the underlying model parameters. This is particularly difficult when the multivariate stochastic process is only partially observed through a 1-dimensional function of its coordinates (the output process), a scenario that we tackle here. Moreover, due to the complexity of SDEs, needed to understand and reproduce the real data, the underlying likelihood is often unknown or intractable. Among several likelihood-free inference approaches, we focus on the simulation-based approximate Bayesian computation (ABC) method. We refer to Marin et al. (2012) and to the recently published book “Handbook of approximate Bayesian computation” for an exhaustive discussion (Sisson et al. 2018).

ABC has become one of the major tools for parameter inference in complex mathematical models in the last decade. The method is based on the idea of deriving an approximate posterior density targeting the true (unavailable) posterior by running massive simulations from the model to replace the intractable likelihood. It was first introduced in the context of population genetics; see, e.g. Beaumont et al. (2002). Since then, it has been successfully applied in a wide range of fields; see, e.g. Barnes et al. (2012), Blum (2010a), Boys et al. (2008), McKinley et al. (2017), Moores et al. (2015) and Toni et al. (2009). Moreover, ABC has also been proposed to infer parameters from time series models (see, e.g. Drovandi et al. 2016; Jasra 2015), state space models (see, e.g. Martin et al. 2019; Tancredi 2019) and SDE models (see, e.g. Kypraios et al. 2017; Maybank et al. 2017; Picchini 2014; Picchini and Forman 2016; Picchini and Samson 2018; Sun et al. 2015; Zhu et al. 2016). Several advanced ABC algorithms have been proposed in the literature, such as sequential Monte Carlo (SMC) ABC, Markov Chain Monte Carlo (MCMC) ABC, sequential annealing ABC, noisy ABC; see, e.g. Fan and Sisson (2018) and the references therein for a recent review. The idea of the basic acceptance–rejection ABC algorithm is to keep a sampled parameter value from the prior as a realisation from the approximate posterior, if the distance between the summary statistics of the synthetic dataset, which is generated conditioned on this parameter value, and the summaries of the original reference data is smaller than some tolerance level. The goal of this paper is to illustrate how building up the ABC method on the structural properties of the underlying SDE, and using a numerical method capable of preserving them in the generation of the data from the model, leads to a successful inference even when applying ABC in this basic acceptance–rejection form.

The performance of any ABC method depends heavily on the choice of “informative enough” summary statistics, a suitable distance measure and a tolerance level $\epsilon$. The quality of the approximation improves as $\epsilon$ decreases, and it has been shown that, under some conditions, the approximated ABC posterior converges to the true one when $\epsilon \rightarrow 0$ (Jasra 2015). At the same time though, the computational cost increases when $\epsilon$ decreases. A possibility is to use ad hoc threshold selection procedures; see, e.g. Barber et al. (2015), Blum (2010b), Lintusaari et al. (2017), Prangle et al. (2014) and Robert (2016). Here, we fix the tolerance level $\epsilon$ as a percentile of the calculated distances. This is another common practice known as “reference table acceptance–rejection ABC”  (Cornuet et al. 2008) and used, for example, in Beaumont et al. (2002), Biau et al. (2015), Sun et al. (2015) and Vo et al. (2015). Instructions for constructing effective summaries and distances are rare, and they depend on the problem under consideration; see, e.g. Fearnhead and Prangle (2012) for a semiautomatic linear regression approach, Jiang et al. (2017) for an automatic construction approach based on training deep neural networks and Blum (2010b) and Prangle (2018) for two recent reviews. To avoid the information loss caused by using non-sufficient summary statistics, another common procedure is to work with the entire dataset; see, e.g. Jasra (2015) and Sun et al. (2015). This requires the application of more sophisticated distances *d* such as the Wasserstein metric (Bernton et al. 2019; Muskulus and Verduyn-Lunel 2011) or other distances designed for time series; for an overview see, e.g. Mori et al. (2016) and the references therein.

When working with stochastic models, simulations from the stochastic simulator, conditionally to the same parameter configuration, yield different trajectories. To consider summary statistics that are less sensitive to the intrinsic stochasticity of the model (Wood 2010), we choose them based on the structural property of an underlying invariant measure. The idea is to map the data, i.e. the realisations of the output process, to an object that is invariant for repeated simulations under the same parameter setting and is instead sensitive to small changes in the parameters. In particular, we map the data to their estimated invariant density and invariant spectral density, taking thus the dependence structure of the dynamical model into account. The distance measure can then be chosen according to the mapped data.

As other simulation-based statistical methods, e.g. MCMC, SMC or machine learning algorithms, ABC relies on the ability of simulating data from the model. However, the exact simulation from complex stochastic models is rarely possible, and thus, numerical methods need to be applied. This introduces a new level of approximation into the ABC framework. When the statistical method is build upon the structural properties of the underlying model, the successful inference can only be guaranteed when these properties are preserved in the synthetic data generated from the model. However, the issue of deriving a property-preserving numerical method when applying ABC to SDEs is usually seen as not so relevant, and it is usually recommended to use the Euler–Maruyama scheme or one of the higher-order approximation methods described in Kloeden and Platen (1992); see, e.g. Picchini (2014), Picchini and Forman (2016), Picchini and Samson (2018) and Sun et al. (2015). In general, these standard methods do not preserve the underlying structural local and global properties of the model; see, e.g. Ableidinger et al. (2017), Malham and Wiese (2013), Moro and Schurz (2007) and Strømmen Melbø and Higham (2004).

Here, we propose to apply structure-preserving numerical splitting schemes within the ABC algorithm. The idea of these methods is to split the SDE into explicitly solvable subequations and to apply a proper composition of the resulting exact solutions. Standard procedures are, for example, the Lie–Trotter method and the usually more accurate Strang approach; see, e.g. Leimkuhler et al. (2016). Since the only approximation enters through the composition of the derived explicit solutions, numerical splitting schemes usually preserve the structural properties of the underlying SDE and accurately reproduce its qualitative behaviour. Moreover, they usually have the same order of convergence as the frequently applied Euler–Maruyama method and are likewise efficient. We refer to Blanes et al. (2009) and Mclachlan and Quispel (2002) for an exhaustive discussion of splitting methods for broad classes of ordinary differential equations (ODEs), which partially have already been carried over to SDEs; see, e.g. Misawa (2001) for a general class of SDEs, Ableidinger and Buckwar (2016) for the stochastic Landau–Lifshitz equations, Bréhier and Goudenège (2019) for the Allen–Cahn equation and Ableidinger et al. (2017) for Hamiltonian type SDEs.

The main contribution of this work lies in the combination of the proposed invariant measure-based summary statistics and the measure-preserving numerical splitting schemes within the ABC framework. We demonstrate that a simulation-based inference method, here ABC, can only perform well if the underlying simulation method preserves the structural properties of the SDE. While the use of preserving splitting schemes within the ABC method yields successful results, applying a general purpose numerical method, such as the Euler–Maruyama discretisation, may result in seriously wrong inferences. We illustrate the proposed spectral density-based and measure-preserving ABC method on the class of stochastic Hamiltonian type equations for which the existence of an underlying unique invariant distribution and measure-preserving numerical splitting schemes have been already intensively studied in the literature; see, e.g. Ableidinger et al. (2017), Mattingly et al. (2002), Leimkuhler and Matthews (2015) and Milstein and Tretyakov (2004). Hamiltonian type SDEs have been investigated in molecular dynamics, where they are typically referred to as Langevin equations; see, e.g. Leimkuhler and Matthews (2015). Recently, they have also received considerable attention in the field of neuroscience as the so-called neural mass models (Ableidinger et al. 2017).

The paper is organised as follows: In Sect. 2, we recall the acceptance–rejection ABC setting. We introduce the invariant measure-based summary statistics and propose a proper distance. We then discuss the importance of considering measure-preserving numerical schemes for the synthetic data generation when exact simulation methods are not applicable and provide a short introduction to numerical splitting methods. In Sect. 3, we introduce Hamiltonian type SDEs and recall two splitting integrators preserving the invariant measure of the model. In Sect. 4, we validate the proposed method by investigating the stochastic harmonic oscillator, for which exact simulation is possible. In Sect. 5, we apply the proposed ABC method to the stochastic Jansen and Rit neural mass model (JR-NMM). We refer to Jansen and Rit (1995) for the original version, an ODE with a stochastic input function, and to Ableidinger et al. (2017) for its reformulation as a Hamiltonian type SDE. This model has been reported to successfully reproduce electroencephalography (EEG) data. We illustrate the performance of the proposed ABC method with both simulated and real data. Final remarks, possible extensions and conclusions are reported in Sect. 6. A supplementary material (Online Resource 1) with further illustrations of the proposed method is available online, and a sample code used to generate the main results is available on github at https://github.com/massimilianotamborrino/sdbmpABC.

## Spectral density-based and measure-preserving ABC for partially observed SDEs with an invariant distribution

Let $(\varOmega ,{\mathcal {F}},{\mathbb {P}})$ be a complete probability space with right continuous and complete filtration ${\mathbb {F}}=\{{\mathcal {F}}\}_{t \in [0,T]}$. Let $\theta \!=\!(\theta _1,\ldots ,\theta _k)$, $k \in {\mathbb {N}}$, be a vector of relevant model parameters. We consider the following *n*-dimensional, $n \in {\mathbb {N}}$, non-autonomous SDE of Itô-type describing the time evolution of a system of interest

1 $$\begin{aligned} \begin{aligned} \hbox {d}X(t)&=f(t,X(t);\theta ) \ \hbox {d}t + {\mathcal {G}} (t, X(t);\theta ) \ \hbox {d}W(t) \\ X(0)&= X_0, \quad t \in [0,T]. \end{aligned} \end{aligned}$$

The initial value $X_0$ is either deterministic or a ${\mathbb {R}}^n$-valued random variable, measurable with respect to ${\mathbb {F}}$. Here, $\mathbf{W }\!=\!(W(t))_{t \in [0,T]}$ is a *r*-dimensional, $r \in {\mathbb {N}}$, Wiener process with independent and ${\mathbb {F}}$-adapted components. We further assume that the drift component $f:~[0,T] \times {\mathbb {R}}^n \rightarrow {\mathbb {R}}^n$ and the diffusion component ${\mathcal {G}}: [0,T] \times {\mathbb {R}}^n \rightarrow {\mathbb {R}}^{n \times r}$ fulfil the necessary global Lipschitz and linear growth conditions, such that the existence and the pathwise uniqueness of an ${\mathbb {F}}$-adapted strong solution process $\mathbf{X }=(X(t))_{t \in [0,T]} \in {\mathbb {R}}^n$ of (1) are guaranteed; see, e.g. Arnold (1974).

We aim to infer the parameter vector $\theta$ inherent in the SDE (1), when the *n*-dimensional solution process $\mathbf{X }$ is only partially observed through the 1-dimensional and parameter-dependent output process

2 $$\begin{aligned} \mathbf{Y }_\theta =(Y_\theta (t))_{t \in [0,T]}=g(\mathbf{X }), \end{aligned}$$

where $g: {\mathbb {R}}^n \rightarrow {\mathbb {R}}$ is a real-valued continuous function of the components of $\mathbf{X }$. Here, we assume that the process $\mathbf{Y }_\theta$ is observed without measurement error, referring to, e.g. Picchini (2014) and Picchini and Forman (2016), where the measurement error is taken into account.

Further, we assume a specific underlying structural model property, namely the existence of a unique invariant measure $\eta _{\mathbf{Y }_\theta }$ on $({\mathbb {R}},{\mathcal {B}}({\mathbb {R}}))$ of the output process $\mathbf{Y }_\theta$, where ${\mathcal {B}}$ denotes the Borel Sigma-algebra. The process has invariant density $f_{\mathbf{Y }_\theta }$ and mean, autocovariance and variance given by

3 $$\begin{aligned} \begin{aligned} {\mathbb {E}}[Y_\theta (t)]&= \eta _{\mu } \in {\mathbb {R}}, \\ \text {Cov}[Y_\theta (t),Y_\theta (s)]&:=r_{\theta }(t,s)=r_{\theta }(t-s),\quad s \le t,\\ \text {Var}[Y_\theta (t)]&=r_{\theta }(0)=\eta _{\sigma ^2} \in {\mathbb {R}}^+. \end{aligned} \end{aligned}$$

If the solution process $\mathbf{X }$ of SDE (1) admits an invariant distribution $\eta _{\mathbf{X }}$ on $({\mathbb {R}}^n,{\mathcal {B}}({\mathbb {R}}^n))$, then the output process $\mathbf{Y }_\theta$ inherits this structural property by means of the marginal invariant distributions of $\eta _{\mathbf{X }}$. Furthermore, if $X(0) \sim \eta _{\mathbf{X }}$, then the process $\mathbf{Y }_\theta =(Y_\theta (t))_{t \in [0,\infty )}$ evolves according to the distribution $\eta _{\mathbf{Y }_{\theta }}$ for all $t \ge 0$. Our goal is to perform statistical inference for the parameter vector $\theta$ of the SDE (1), when the solution process **X** is partially observed through discrete time measurements of the output process $\mathbf{Y }_\theta$ given in (2), by benefiting from the (in general unknown) invariant distribution $\eta _{\mathbf{Y }_\theta }$ satisfying (3).

### The ABC method

Let $y=(y(t_i))_{i=1}^l$, $l \in {\mathbb {N}}$, be the reference data, corresponding to discrete time observations of the output process $\mathbf{Y }_\theta$. Let us denote by $\pi (\theta )$ and $\pi (\theta |y)$ the prior and the posterior density, respectively. For multivariate complex SDEs, the underlying likelihood is often unknown or intractable. The idea of the ABC method is to derive an approximate posterior density for $\theta$ by replacing the unknown likelihood by many simulations of synthetic datasets from the underlying model (1) that are mapped to $\mathbf{Y}_\theta$ through (2). The basic acceptance–rejection ABC algorithm consists of the following three steps: i. Sample a value $\theta '$ from the prior $\pi (\theta )$; ii. Conditionally on $\theta '$, simulate a new artificial dataset from the model (1) and derive the synthetic data $y_{\theta '}=(y_{\theta '}(t_i))_{i=0}^m, t_0=0,t_m=T,m\in {\mathbb {N}}$, from the process $\mathbf{Y}_{\theta '}$ given by (2); iii. Keep the sampled parameter value $\theta '$ as a realisation from an ABC posterior if the distance $d(\cdot )$ between a vector of summary statistics $s=(s_1,\ldots , s_h), h \in {\mathbb {N}}$, of the original and the synthetic data is smaller than some threshold level $\epsilon \ge 0$, i.e. $d(s(y),s(y_{\theta '}))<\epsilon$. When $\epsilon =0$ and *s* is a vector of sufficient statistics for $\theta$, the acceptance–rejection ABC produces samples from the true posterior $\pi (\theta |y)$. Due to the complexity of the underlying SDE (1), we cannot derive non-trivial sufficient statistics *s* for $\theta$. Moreover, due to the underlying stochasticity of the model, ${\mathbb {P}}(d(s(y),s(y_{\theta '}))=0)=0$. Thus, $\epsilon$ is required to be strictly positive.
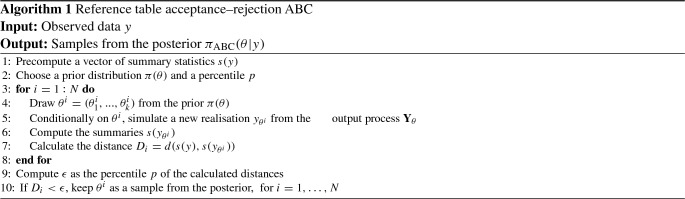


Throughout, we set $\epsilon$ a posteriori, in the spirit of the reference table acceptance–rejection ABC (Cornuet et al. 2008), summarised in Algorithm 1, that is, we first produce the reference table $(\theta ^i,D_i)$, $i=1,\ldots ,N$, and then obtain $\epsilon$ as a percentile *p* of the calculated distances $D_i$. Algorithm 1 yields samples from an approximated posterior $\pi _{\text {ABC}}(\theta |y)$ according to

$$\begin{aligned} \pi (\theta |y) \approx \pi _{\text {ABC}} (\theta |y) = \pi \{ \theta | d(s(y),s(y_\theta ))<\epsilon \}. \end{aligned}$$

**Fig. 1:**
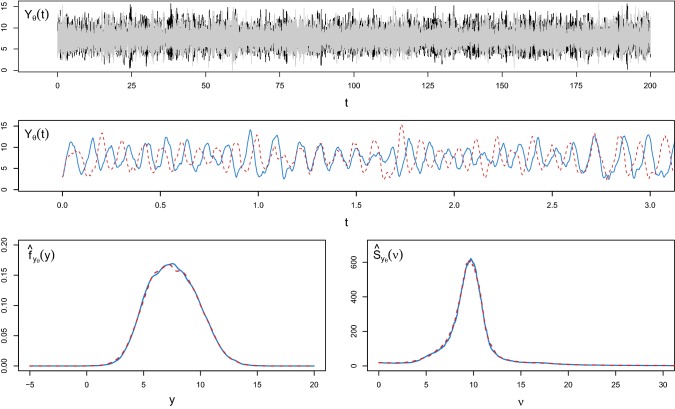
Two realisations of the output process of the stochastic JR-NMM (25) generated with the numerical splitting method (17) for an identical choice of $\theta$. The lengths of the time intervals are $T=200$ and $T=3$ (to provide a zoom) in the top and middle panel, respectively. The two invariant densities and two invariant spectral densities, estimated from the two full datasets shown in the top panel, are reported in the lower panel on the left and right, respectively

Besides the tolerance level $\epsilon$, the quality of the ABC method depends strongly on the choice of suitable summary statistics combined with a proper distance measure and on the numerical method used to generate the synthetic data from the model. In the following, we introduce summaries that are very effective for the class of models having an underlying invariant distribution, we suggest a proper distance based on them, and we propose the use of measure-preserving numerical splitting schemes.

### An effective choice of summaries and distances: spectral density-based ABC

When applying ABC to stochastic models, an important statistical challenge arises. Due to the intrinsic randomness, repeated simulations of the process $\mathbf{Y }_\theta$ under the same parameter vector $\theta$ may yield very different trajectories. An illustration is given in Fig. 1 (top and middle panels), where we report two trajectories of the output process of the stochastic JR-NMM (25) generated with an identical parameter configuration. This model is a specific SDE of type (1), observed through $\mathbf{Y }_\theta$ as in (2), and admitting an invariant distribution $\eta _{\mathbf{Y }_{\theta }}$ satisfying (3). See Sect. 5 for a description of the model. In the top panel, we visualise the full paths for a time $T=200$, while in the middle panel we provide a zoom, showing only the initial part.

#### Proposal 1

To use the property of an invariant measure $\eta _{{{\mathbf{Y}}}_\theta }$ and to map the data $y_\theta$ to their estimated invariant density ${\hat{f}}_{y_\theta }$ and invariant spectral density ${\hat{S}}_{y_\theta }$.

Instead of working with the output process $\mathbf{Y }_\theta$, we take advantage of the structural model property $\eta _{\mathbf{Y }_{\theta }}$ and focus on its invariant density $f_{\mathbf{Y }_\theta }$ and its invariant spectral density $S_{\mathbf{Y }_\theta }$. Both are deterministic functions characterised by the underlying parameters $\theta$ and thus invariant for repeated simulations under the same parameter configuration. The invariant spectral density is obtained from the Fourier transformation of the autocovariance function $r_{\theta }$, and it is given by

4 $$\begin{aligned} S_{\mathbf{Y }_\theta }={\mathcal {F}}\{ r_\theta \} (\omega )=\int _{-\infty }^{\infty } r_{\theta }(\tau )\hbox {e}^{-{{\mathrm{i}}}\omega \tau } \ \hbox {d}\tau , \end{aligned}$$

for $\omega \in [-\pi ,\pi ]$. The angular frequency $\omega$ relates to the ordinary frequency $\nu$ via $\omega =2\pi \nu$. Since both $f_{\mathbf{Y }_\theta }$ and $S_{\mathbf{Y }_\theta }$ are typically unknown, we estimate them from a dataset $y_\theta$. First, we estimate the invariant density $f_{\mathbf{Y }_\theta }$ with a kernel density estimator, denoted by ${\hat{f}}_{y_\theta }$; see, e.g. Pons (2011). Second, we estimate the invariant spectral density $S_{\mathbf{Y }_\theta }$ (4) with a smoothed periodogram estimator (Cadonna et al. 2017; Quinn et al. 2014), denoted by ${\hat{S}}_{y_\theta }$, which is typically evaluated at Fourier frequencies. Differently from the invariant density, the invariant spectral density does not account for the mean ${\mathbb {E}}[\mathbf{Y }_\theta ]$ but captures the dependence structure of the data coming from the model. We define the invariant measure-based summary statistics *s* of a dataset $y_\theta$ as

5 $$\begin{aligned} s(y_\theta ):=({\hat{S}}_{y_\theta },{\hat{f}}_{y_\theta }). \end{aligned}$$

Figure 1 shows the two estimated invariant densities (left lower panel) and invariant spectral densities (right lower panel), all derived from the full paths of the output process $\mathbf{Y }_\theta$ (top panel).

After performing the data mapping (5), which significantly reduces the randomness in the output of the stochastic simulator, the distance $d(\cdot )$ can be chosen among the distance measures between two ${\mathbb {R}}$-valued functions. Here, we consider the integrated absolute error (IAE) defined by

6 $$\begin{aligned} \text {IAE}(g_1,g_2):=\int \limits _{{\mathbb {R}}} \ \Bigl |g_1(x)-g_2(x)\Bigr | \ \hbox {d}x \in {\mathbb {R}}^+. \end{aligned}$$

Another natural possibility could be a distance chosen among the so-called *f*-divergences (see, e.g. Sason and Verdú 2016), or the Wasserstein distance, recently proposed for ABC (Bernton et al. 2019). Within the ABC framework (see Step 7 in Algorithm 1), we suggest to use the following distance

7 $$\begin{aligned} \hbox {d}(s(y),s(y_\theta )):= \text {IAE}({\hat{S}}_{y},{\hat{S}}_{y_\theta })+w \cdot \text {IAE}({\hat{f}}_{y},{\hat{f}}_{y_\theta }), \end{aligned}$$

returning a weighted sum of the areas between the densities estimated from the original and the synthetic datasets. Here, $w \ge 0$ is a weight that we assign to the part related to the IAE of the invariant densities such that the two errors are of the same “order of magnitude”. This is particularly needed because, differently from the invariant density, the invariant spectral density does not integrate to 1. We obtain a value for the weight by performing an ABC pilot simulation. It consists in reiterating the following steps *L* times:
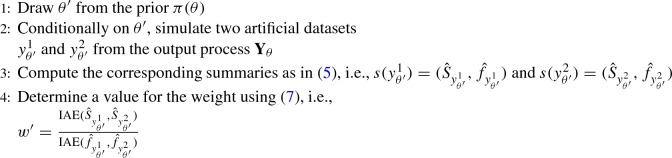


Then, we take the median of the resulting *L* values $w'$. See, e.g. Prangle (2017) for alternative approaches for the derivation of weights among summary statistics. Since the densities ${{\hat{f}}}_{{y_\theta }}$ and ${{\hat{S}}}_{y_\theta }$ are estimated at discrete points, the IAE (6) is approximated applying trapezoidal integration.

In our applications we consider $M \in {\mathbb {N}}$ realisations of the output process $\mathbf{Y }_\theta$ sampled at $l \in {\mathbb {N}}$ discrete points in time, resulting in observed data arranged as a matrix $y \in {\mathbb {R}}^{M \times l}$. Under this experimental scenario, the median of the distances (7) computed for each of the *M* datasets

8 $$\begin{aligned} D= \text {median}\left\{ \left( \text {IAE}({\hat{S}}_{y_k},{\hat{S}}_{{y}_{\theta }}) + w \cdot \text {IAE}({\hat{f}}_{y_k},{\hat{f}}_{{y}_{\theta }}) \right) _{k=1}^M \right\} \nonumber \\ \end{aligned}$$

is then returned as a global distance in Step 7 of Algorithm 1. Other strategies can be adopted. For example, considering the mean instead yields similar results in all our experiments. One can interpret *y* as a long-time trajectory (when using simulated observed reference data) or as a long-time recording of the modelled phenomenon (when using real observed reference data) that is cut into *M* pieces. Alternatively, *y* would consist of M independent repeated experiments or simulations, when dealing with real or simulated data, respectively. As expected, having $M>1$ datasets improves the quality of the estimation due to the increased number of observations.

### A new proposal of synthetic data generation: measure-preserving ABC

A crucial aspect of ABC and of all other simulation-based methods is the ability of simulating from the model (Step 5 of Algorithm 1). Consider a discretised time grid with the equidistant time step $\varDelta =t_{i+1}-t_{i}$, and let ${\tilde{y}}_\theta =({\tilde{y}}_\theta (t_i))_{i=1}^m$ be a realisation from the output process $\widetilde{\mathbf{Y }}_{\theta } =({\widetilde{Y}}_\theta (t_i))_{i=1}^m$, obtained through a numerical method, approximating $\mathbf{Y }_\theta$ at the discrete data points, i.e. ${\widetilde{Y}}_\theta (t_i) \approx Y_\theta (t_i)$. The lack of exact simulation schemes, i.e. ${\widetilde{Y}}_\theta (t_i) = Y_\theta (t_i)$, introduces a new level of approximation in the statistical inference. In particular, Algorithm 1 samples from an approximated posterior density of the form

$$\begin{aligned} \pi (\theta |y) \approx \pi ^{\text {num}}_{\text {ABC}} (\theta |y) := \pi \{ \theta | d(s(y),s({{\tilde{y}}}_\theta ))<\epsilon \}. \end{aligned}$$

As a consequence, $y_\theta$ in Step 5 of Algorithm 1 is replaced by its numerical approximation ${\tilde{y}}_\theta$.

The commonly used Euler–Maruyama scheme yields discretised trajectories of the solution process $\mathbf{X }$ of the SDE (1) through (Kloeden and Platen 1992)

9 $$\begin{aligned} {\widetilde{X}}(t_{i+1})={\widetilde{X}}(t_i)+f(t_i,{\widetilde{X}}(t_i);\theta ) \varDelta + {\mathcal {G}}(t_i,{\widetilde{X}}(t_i);\theta ) \xi _i,\nonumber \\ \end{aligned}$$

where $\xi _i$ are Gaussian vectors with null mean and variance $\varDelta {\mathbb {I}}_n$, where ${\mathbb {I}}_n$ denotes the $n \times n$-dimensional identity matrix. As previously discussed, in general, the Euler–Maruyama method does not preserve the underlying invariant distribution $\eta _{{{\mathbf{Y}}}_\theta }$.

#### Proposal 2

To adopt a numerical method for the synthetic data generation that preserves the underlying invariant measure of the model.

**Fig. 2:**
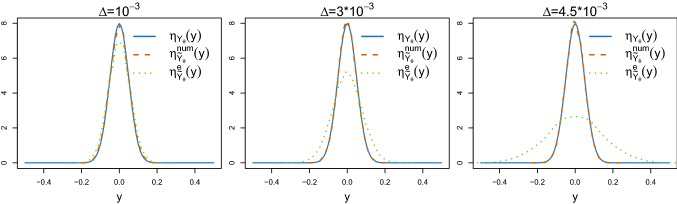
Comparison of the true invariant density of the weakly damped stochastic harmonic oscillator (23) (blue solid lines) with the densities estimated using a kernel density estimator applied on data $y_\theta$ generated by the measure-preserving splitting scheme (22) (orange dashed lines) and the Euler–Maruyama method (9) (green dotted lines) with time step $\varDelta$ up to time $T=10^3$. The values of the time steps are $\varDelta =10^{-3}$ (left panel), $3\cdot 10^{-3}$ (central panel) and $4.5 \cdot 10^{-3}$ (right panel), respectively. (Color figure online)

We apply numerical splitting schemes within the ABC framework and provide a brief account of their theory. Let us assume that the drift *f* and the diffusion ${\mathcal {G}}$ of SDE (1) can be written as

$$\begin{aligned} f(t,X(t);\theta )= & {} \sum _{j=1}^{d} f^{[j]}(t,X(t);\theta ), \\ {\mathcal {G}}(t,X(t);\theta )= & {} \sum _{j=1}^{d} {\mathcal {G}}^{[j]}(t,X(t);\theta ), \quad d \in {\mathbb {N}}. \end{aligned}$$

The goal is to decompose *f* and ${\mathcal {G}}$ in a way such that the resulting *d* subequations

$$\begin{aligned} \hbox {d}X(t)=f^{[j]}(t,X(t);\theta ) \ \hbox {d}t + {\mathcal {G}}^{[j]} (t, X(t);\theta ) \ \hbox {d}W(t), \end{aligned}$$

for $j \in \{ 1,\ldots ,d \}$, can be solved exactly. Note that, the terms ${\mathcal {G}}^{[j]}$ can be null, resulting in deterministic equations (ODEs). Let $X^{[j]}(t)=\varphi _t^{[j]}(X_0)$ denote the exact solutions (flows) of the above subequations at time *t* and starting from $X_0$. Once these explicit solutions are derived, a proper composition needs to be applied. Here we use the Strang approach (Mclachlan and Quispel 2002; Strang 1968)

$$\begin{aligned} \left( \varphi _{\varDelta /2}^{[1]} \circ \cdots \circ \varphi _{\varDelta /2}^{[d-1]} \circ \varphi _{\varDelta }^{[d]} \circ \varphi _{\varDelta /2}^{[d-1]} \circ \cdots \circ \varphi _{\varDelta /2}^{[1]} \right) (x), \end{aligned}$$

$x \in {\mathbb {R}}^n$, that provides a numerical solution for the original SDE (1).

In Fig. 2, we illustrate how the numerical splitting method preserves the underlying invariant measure of the weakly damped stochastic harmonic oscillator (23), independently from the choice of the time step $\varDelta$. This is a specific SDE of type (1), observed through $\mathbf{Y }_\theta$ as in (2) and with a known invariant distribution $\eta _{\mathbf{Y }_{\theta }}$. See Sect. 3 for the detailed numerical splitting scheme and Sect. 4 for a description of the model. In contrast, the Euler–Maruyama scheme performs worse as $\varDelta$ increases. Each subplot shows a comparison of the true invariant density (blue solid lines) and the corresponding kernel estimate ${\hat{f}}_{y_\theta }$ based on a path $y_\theta$ from the model, generated from the measure-preserving numerical splitting scheme (22) (orange dashed lines) or the Euler–Maruyama approach (green dotted lines). The data are generated under $T=10^3$ and different values for the time step, namely $\varDelta =10^{-3}$, $3\cdot 10^{-3}$, $4.5\cdot 10^{-3}$.

### Notation

We apply the summary statistics (5) and the distance (8) in Algorithm 1. We use the notation Algorithm 1 (i) [spectral density-based ABC method] when the synthetic data are simulated exactly, Algorithm 1 (ii) [spectral density-based and measure-preserving ABC method] when a measure-preserving numerical splitting scheme is applied and Algorithm 1 (iii) when we generate the data with the non-preserving Euler–Maruyama scheme.

To evaluate the performance of the proposed ABC method, we analyse the marginal posterior densities, denoted by $\pi _{\text {ABC}}^*(\theta _j|y)$, $j \in \{1,\ldots ,k\}$, obtained from the posterior density $\pi _{\text {ABC}}^*(\theta |y)$ corresponding to $\pi _{\text {ABC}}(\theta |y)$, $\pi _{\text {ABC}}^\text {num}(\theta |y)$ or $\pi _{\text {ABC}}^e(\theta |y)$, depending on whether we obtain it from Algorithm 1 (i), (ii) or (iii). Following this notation, we define by ${\hat{\theta }}_{\text {ABC},j}^*$ the marginal ABC posterior means.

## An illustration on Hamiltonian type SDEs

We illustrate the proposed ABC approach on Hamiltonian type SDEs and define the *n*-dimensional ($n=2d$, $d\in {\mathbb {N}}$) stochastic process

$$\begin{aligned} \mathbf{X }:=(\mathbf{Q },\mathbf{P })^{\prime }=(Q(t),P(t))^{\prime }_{t \in [0,T]}, \end{aligned}$$

consisting of the two *d*-dimensional components

$$\begin{aligned} \mathbf{Q }=({\mathbf {X}}_{{\mathbf {1}}},\ldots ,\mathbf {X_d})^{\prime } \quad \text {and} \quad \mathbf{P }=(\mathbf {X_{d+1}},\ldots ,\mathbf {X_{2d}})^{\prime }, \end{aligned}$$

where $^{\prime }$ denotes the transpose. The *n*-dimensional SDE of Hamiltonian type with initial value $X_0=~(Q_0,P_0)^{\prime }$ and *d*-dimensional ($r=d$) Wiener process $\mathbf{W }$ describes the time evolution of $\mathbf{X }$ by

10 $$\begin{aligned} \begin{aligned}&\hbox {d} \underbrace{ \begin{pmatrix} Q(t) \\ P(t) \end{pmatrix}}_{X(t)} = \underbrace{ \begin{pmatrix} {\mathbb {O}}_d \\ \varSigma _\theta \end{pmatrix}}_{{\mathcal {G}}(\theta )} \hbox {d}W(t) \\&+ \,\underbrace{ \begin{pmatrix} \nabla _P H(Q(t),P(t)) \\ -\nabla _Q H(Q(t),P(t)) -2\varGamma _\theta P(t)+G(Q(t);\theta ) \end{pmatrix}}_{f(X(t);\theta )} \hbox {d}t.\end{aligned} \end{aligned}$$

We denote with ${\mathbb {O}}_d$ the $d \times d$-dimensional zero matrix and with $\nabla _Q$ and $\nabla _P$ the gradient with respect to *Q* and *P*, respectively. The SDE (10) consists of 4 parts, each representing a specific type of behaviour. In this configuration, the first is the *Hamiltonian part* involving $H:{\mathbb {R}}^d \times {\mathbb {R}}^d \rightarrow {\mathbb {R}}_0^+$ given by

$$\begin{aligned} H(\mathbf{Q },\mathbf{P }):=\frac{1}{2}(\left||\mathbf{P }\right||^2_{{\mathbb {R}}^d}+\left||\varLambda _\theta \mathbf{Q }\right||^2_{{\mathbb {R}}^d}), \end{aligned}$$

where $\varLambda _\theta =\text {diag}[\lambda _{1},\ldots ,\lambda _{d}] \in {\mathbb {R}}^{d \times d}$ is a diagonal matrix. The second is the *linear damping part*, described by the diagonal matrix $\varGamma _\theta =\text {diag}[\gamma _{1},\ldots ,\gamma _{d}] \in {\mathbb {R}}^{d \times d}$. The third is the *nonlinear displacement part*, consisting of the nonlinear and globally Lipschitz continuous function $G: {\mathbb {R}}^d \rightarrow {\mathbb {R}}^d$. The fourth corresponds to the *diffusion part*, given by $\varSigma _\theta =\text {diag}[\sigma _{1},\ldots ,\sigma _{d}] \in {\mathbb {R}}^{d \times d}$.

### Structural model property

Under the requirement of non-degenerate matrices $\varLambda _\theta$, $\varGamma _\theta$ and $\varSigma _\theta$, i.e. strictly positive diagonal entries, Hamiltonian type SDEs as in (10) are often ergodic. As a consequence, the distribution of the solution process $\mathbf{X }$ (and thus of the output process $\mathbf{Y}_\theta$) converges exponentially fast towards a unique invariant measure $\eta _{\mathbf{X }}$ on $({\mathbb {R}}^n,{\mathcal {B}}({\mathbb {R}}^n))$ (and thus $\eta _{{{\mathbf{Y}}}_\theta }$ on $({\mathbb {R}},{\mathcal {B}}({\mathbb {R}}))$; see, e.g. Ableidinger et al. (2017) and the references therein.

### Measure-preserving numerical splitting schemes

Two splitting approaches for SDE (10) are provided, see Ableidinger et al. (2017). Due to the nonlinear term *G*, the SDE (10) cannot be solved explicitly. With the purpose of excluding *G*, the Hamiltonian type SDE (10) is split into the two subsystems

11 $$\begin{aligned}&\hbox {d}\begin{pmatrix} Q(t)\\ P(t) \end{pmatrix}=\underbrace{ \left( \begin{array}{c}{\mathbb {O}}_d\\ \varSigma _\theta \end{array} \right) }_{{\mathcal {G}}^{[1]}(\theta )} \hbox {d}W(t) \nonumber \\&+\,\underbrace{\begin{pmatrix} \nabla _P H((t),P(t)) \\ -\nabla _Q H(Q(t),P(t)) - 2 \varGamma _\theta P(t) \end{pmatrix}}_{f^{[1]}(X(t);\theta )}\hbox {d}t, \end{aligned}$$

12 $$\begin{aligned}&\hbox {d}\begin{pmatrix} Q(t) \\ P(t) \end{pmatrix}=\underbrace{\begin{pmatrix} 0_d \\ G(Q(t);\theta ) \end{pmatrix}}_{f^{[2]}(Q(t);\theta )}\hbox {d}t, \end{aligned}$$

where $0_d$ denotes the *d*-dimensional zero vector. This results in a linear SDE with additive noise (11) and a nonlinear ODE (12) that can be both explicitly solved. Indeed, since $\nabla _P H(Q(t),P(t))=P(t)$ and $\nabla _Q H(Q(t),P(t))\!=\!\varLambda _\theta ^2 Q(t)$, Subsystem (11) can be rewritten as

13 $$\begin{aligned} \hbox {d}X(t)=A \cdot X(t) \ \hbox {d}t+B \ \hbox {d}W(t), \quad t \ge 0, \end{aligned}$$

with $$A= \begin{pmatrix} {\mathbb {O}}_d &{} \quad {\mathbb {I}}_d\\ -\varLambda _\theta ^2 &{} \quad -2\varGamma _\theta \end{pmatrix} $$ and $$B=\left( \begin{array}{c}{\mathbb {O}}_d\\ \varSigma _\theta \end{array} \right) $$. The exact path of System (13) is obtained through (Arnold 1974)

14 $$\begin{aligned} X(t_{i+1})=\hbox {e}^{A \varDelta } \cdot X(t_{i}) + \xi _i, \end{aligned}$$

where $\xi _i$ are *n*-dimensional Gaussian vectors with null mean and variance $C(\varDelta )$, where the matrix *C*(*t*) follows the dynamics of the matrix-valued ODE

15 $$\begin{aligned} {\dot{C}}(t)=AC(t)+C(t)A^{\prime } + BB^{\prime }, \quad C(0)={\mathbb {O}}_n. \end{aligned}$$

Moreover, since the nonlinear term *G* depends only on the component $\mathbf{Q }$, the exact path of Subsystem (12) is obtained through

16 $$\begin{aligned} X(t_{i+1})=X(t_i) + \left( \begin{array}{c} 0_d\\ \varDelta G(Q(t_i);\theta ) \end{array} \right) . \end{aligned}$$

We apply the Strang approach given by

17 $$\begin{aligned} (\varphi ^b_{\varDelta /2} \circ \varphi ^a_{\varDelta } \circ \varphi ^b_{\varDelta /2})(x), \quad x \in {\mathbb {R}}^n, \end{aligned}$$

where $\varphi _t^a$ and $\varphi _t^b$ denote the exact solutions (14) and (16) of (11) and (12), respectively. Hence, given $X(t_i)$, we obtain the next value $X(t_{i+1})$ by applying the following three steps:
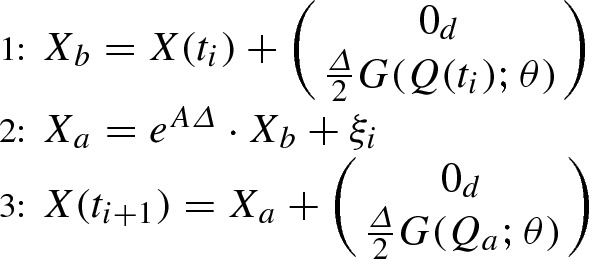


The derivation of the subsystems is not unique. For example, another possibility is to combine the stochastic term with the nonlinear part, yielding the subsystems

18 $$\begin{aligned} \hbox {d}\begin{pmatrix} Q(t)\\ P(t) \end{pmatrix}= & {} \underbrace{\begin{pmatrix} \nabla _P H(Q(t),P(t)) \\ -\nabla _Q H(Q(t),P(t)) - 2 \varGamma _\theta P(t) \end{pmatrix}}_{f^{[1]}(X(t);\theta )}\hbox {d}t, \end{aligned}$$

19 $$\begin{aligned} \hbox {d}\begin{pmatrix} Q(t) \\ P(t) \end{pmatrix}= & {} \underbrace{\begin{pmatrix} 0_d \\ G(Q(t);\theta ) \end{pmatrix}}_{f^{[2]}(Q(t);\theta )}\hbox {d}t+ \underbrace{\left( \begin{array}{c}{\mathbb {O}}_d\\ \varSigma _\theta \end{array} \right) }_{{\mathcal {G}}^{[2]}(\theta )}\hbox {d}W(t). \end{aligned}$$

The exact path of (18) is given by

20 $$\begin{aligned} X(t_{i+1})=\hbox {e}^{A \varDelta } \cdot X(t_{i}), \end{aligned}$$

while the exact path of (19) is obtained through

21 $$\begin{aligned} X(t_{i+1})=\left( \begin{array}{c} Q({t_{i}})\\ P({t_{i}})+\varDelta G(Q(t_i);\theta ) + \varSigma _\theta \cdot \xi _i \end{array} \right) , \end{aligned}$$

where $\xi _i$ are *d*-dimensional Gaussian vectors with null mean and variance $\varDelta {\mathbb {I}}_d$. The Strang approach is now given by

22 $$\begin{aligned} (\varphi ^c_{\varDelta /2} \circ \varphi ^d_{\varDelta } \circ \varphi ^c_{\varDelta /2})(x), \quad x \in {\mathbb {R}}^n, \end{aligned}$$

where $\varphi _t^c$ and $\varphi _t^d$ denote the exact solutions (20) and (21) of (18) and (19), respectively. Thus, given $X(t_i)$, the next value $X(t_{i+1})$ is obtained via:
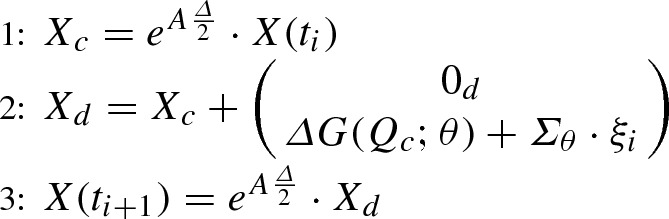


### Implementation details

The ABC procedure is coded in the computing environment **R** (R Development Core Team 2011), using the package **Rcpp** (Eddelbuettel and François 2011), which offers a seamless integration of **R** and C++, drastically reducing the computational time of the algorithms. The code is then parallelised using the R-packages foreach and doParallel, taking advantage of the *for loop* in the algorithm. All simulations are run on the HPC cluster RADON1, a high-performing multiple core cluster located at the Johannes Kepler University Linz. To obtain smoothed periodogram estimates, we apply the R-function spectrum. It requires the specification of a smoothing parameter span. In all our experiments, we use span  $=5T$. In addition, we avoid using a logarithmic scale by setting the log parameter to “no”. To obtain kernel estimates of the invariant density, we apply the R-function density. Here, we use the default value for the smoothing bandwidth bw and set the number of points at which the invariant density has to be estimated to n$=10^3$. The invariant spectral density is estimated at the default values of the spectrum function. A sample code is publicly available on github at https://github.com/massimilianotamborrino/sdbmpABC.

## Validation of the proposed ABC method when exact simulation is possible

In this section, we illustrate the performance of the proposed ABC approach on a model problem (weakly damped stochastic harmonic oscillator) of Hamiltonian type (10) with vanishing nonlinear displacement term $G \equiv 0$. Linear SDEs of this type reduce to (13) and allow for an exact simulation of sample paths through (14). Therefore, we can apply the spectral density-based ABC Algorithm 1 (i) under the optimal condition of exact and thus $\eta _{\mathbf{Y }_\theta }$-preserving data generation. Its performance is illustrated in Sect. 4.2. To investigate how the numerical error in the synthetic data generation impinges on the ABC performance, in Sect. 4.3 we compare $\pi _{\text {ABC}}(\theta |y)$ with the posterior densities $\pi _{\text {ABC}}^{\text {num}}(\theta |y)$ and $\pi _{\text {ABC}}^{\text {e}}(\theta |y)$ obtained from Algorithm 1 (ii) and (iii) using the measure-preserving numerical splitting scheme (22) and the non-preserving Euler–Maruyama method (9), respectively.

**Fig. 3:**
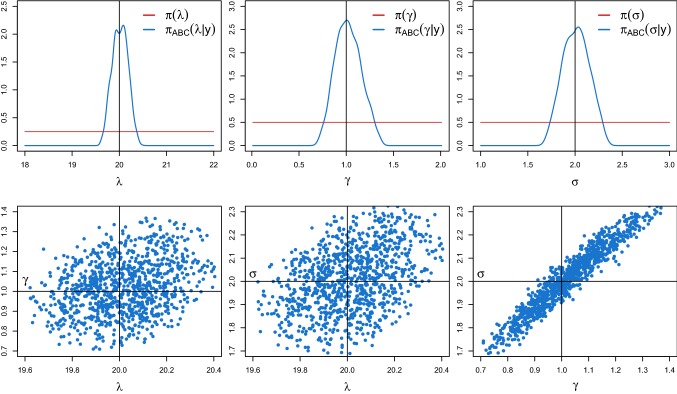
Top panels: ABC marginal posterior densities $\pi _{\text {ABC}}(\theta _j|y)$ (blue lines) of $\theta =(\lambda ,\gamma ,\sigma )$ of the weakly damped stochastic harmonic oscillator (23) and uniform priors (red lines). The posteriors are obtained from Algorithm 1 (i). The vertical lines represent the true parameter values. Lower panels: Pairwise scatterplots of the kept ABC posterior samples. (Color figure online)

### Weakly damped stochastic harmonic oscillator: the model and its properties

We investigate the 2-dimensional Hamiltonian type SDE

23 $$\begin{aligned} \hbox {d} \begin{pmatrix} Q(t) \\ P(t) \end{pmatrix} = \begin{pmatrix} P(t) \\ -\lambda ^2Q(t) -2\gamma P(t) \end{pmatrix} \hbox {d}t \ + \ \begin{pmatrix} 0 \\ \sigma \end{pmatrix} \hbox {d}W(t),\nonumber \\ \end{aligned}$$

with strictly positive parameters $\gamma$, $\lambda$ and $\sigma$. Depending on the choice of $\gamma$ and $\lambda$, (23) models different types of harmonic oscillators, which are common in nature and of great interest in classical mechanics. Here, we focus on the weakly damped harmonic oscillator, satisfying the condition $\lambda ^2-\gamma ^2>0$. Our goal is to estimate $\theta =(\lambda ,\gamma ,\sigma )$ assuming that the solution process $\mathbf{X }=(\mathbf{Q },\mathbf{P })^{\prime }$ is partially observed through the first coordinate, i.e. $\mathbf{Y }_\theta =\mathbf{Q }$. An illustration of the performance of Algorithm 1 (i) for the critically damped case satisfying $\lambda ^2-\gamma ^2=0$, when only the second coordinate is observed, is reported in the supplementary material. The solution process $\mathbf{X }$ of SDE (23) is normally distributed according to

$$\begin{aligned} X(t) \sim \eta _\mathbf{X }(t):= {\mathcal {N}}\Big (\hbox {e}^{At} \cdot {\mathbb {E}}[X_0], \ \text {Var}[\hbox {e}^{At} \cdot X_0] + C(t)\Big ), \end{aligned}$$

with *A* and *C* introduced in (13) and (15), respectively. The invariant distribution $\eta _\mathbf{X }$ of the solution process $\mathbf{X }$ is given by

$$\begin{aligned} \eta _\mathbf{X }=\lim \limits _{t \rightarrow \infty } \eta _\mathbf{X }(t)= {\mathcal {N}}\left( \begin{pmatrix} 0 \\ 0 \end{pmatrix},\begin{pmatrix} \frac{\sigma ^2}{4\gamma \lambda ^2} &{} \quad 0 \\ 0 &{}\quad \frac{\sigma ^2}{4\gamma } \end{pmatrix}\right) . \end{aligned}$$

Consequently, the structural property $\eta _{\mathbf{Y }_\theta }$ of the output process $\mathbf{Y }_\theta$ becomes

24 $$\begin{aligned} \eta _{\mathbf{Y }_\theta } = {\mathcal {N}}\left( 0,\frac{\sigma ^2}{4\gamma \lambda ^2}\right) , \end{aligned}$$

and the stationary dependency is captured by the autocovariance function

$$\begin{aligned} r_\theta (\varDelta ) = \frac{\sigma ^2}{4\lambda ^2}\hbox {e}^{-\gamma \varDelta } \left[ \frac{1}{\gamma }\cos (\kappa \varDelta )+\frac{1}{\kappa }\sin (\kappa \varDelta )\right] , \end{aligned}$$

where $$\kappa =\sqrt{\lambda ^2-\gamma ^2}$$.

### Validation of the spectral density-based ABC Algorithm 1 (i)

To compare the performances of Algorithms 1 (i)–(iii) on the same data, we consider the same $M=10$ observed paths simulated with the exact scheme (14), using a time step $\varDelta =10^{-2}$ over a time interval of length $T=10^3$. As true parameters for the simulation of the reference data, we choose

$$\begin{aligned} \theta ^t=(\lambda ^t, \gamma ^t,\sigma ^t)=(20,1,2). \end{aligned}$$

We use the exact simulation scheme (14) to generate $N=2\cdot 10^6$ synthetic datasets in [0, *T*] and with the same time step as the observed data. We choose independent uniform priors, in particular,

$$\begin{aligned} \lambda \sim U(18,22), \qquad \gamma \sim U(0.01,2.01), \qquad \sigma \sim U(1,3). \end{aligned}$$

The tolerance level $\epsilon$ is chosen as the $0.05{\text {th}}$ percentile of the calculated distances. Hence, we keep $10^3$ of all the sampled values for $\theta$. In all the considered examples (see also the supplementary material), the performance of the ABC algorithms for the estimation of the parameters of SDE (23) does not improve when incorporating the information of the invariant densities into the distance (7). This is because the mean of the invariant distribution (24) is zero. Hence, to reduce the computational cost, we set $w=0$ and base our distance only on the invariant spectral density, estimated by the periodogram.

Figure 3 (top panels) shows the marginal ABC posterior densities $\pi _{\text {ABC}}(\theta _j|y)$ (blue lines) and their flat uniform priors $\pi (\theta _j)$ (red lines). The proposed ABC Algorithm 1 (i) provides marginal posterior densities centred around the true values $\theta ^t$, represented by the black vertical lines. The posterior means are given by

$$\begin{aligned} ({\hat{\lambda }}_{\text {ABC}},{\hat{\gamma }}_{\text {ABC}},{\hat{\sigma }}_{\text {ABC}})=(20.015,1.022,2.011). \end{aligned}$$

In the lower panels of Fig. 3, we report the pairwise scatterplots of the kept ABC posterior samples. Note that, since the kept values of $\lambda$ are uncorrelated with those of the other parameters, the support of the obtained marginal posterior density is approximately the same as when estimating only $\theta =\lambda$ or $\theta =(\lambda ,\gamma )$ (cf. supplementary material). Vice versa, since the kept ABC posterior samples of the parameters $\gamma$ and $\sigma$ are correlated, the support of $\pi _{\text {ABC}}(\gamma |y)$ is larger than that obtained when estimating $\theta =(\lambda ,\gamma )$. Despite this correlation, Algorithm 1 (i) allows for a successful inference of all the three parameters.

### Validation of the spectral density-based and measure-preserving ABC Algorithm 1 (ii)

**Fig. 4:**
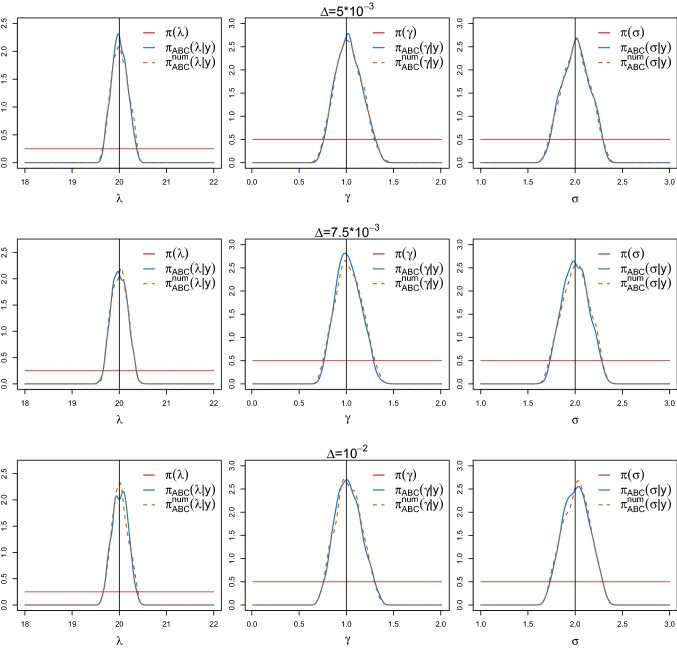
ABC marginal posterior densities of $\theta =(\lambda ,\gamma ,\sigma )$ of the weakly damped stochastic harmonic oscillator (23) obtained from Algorithm 1 (i) with the exact simulation method (14) (blue solid lines) and Algorithm 1 (ii) combined with the splitting scheme (22) (orange dashed lines) for different choices of the time step $\varDelta$. In particular, $\varDelta = 5\cdot 10^{-3}$ (top panels), $7.5 \cdot 10^{-3}$ (middle panels) and $10^{-2}$ (lower panels). The red horizontal lines denote the uniform priors and the black vertical lines the true parameter values. (Color figure online)

In Fig. 4, we report the approximated marginal posteriors $\pi _{\text {ABC}}(\theta _j|y)$ (blue solid lines) and $\pi _{\text {ABC}}^\text {num}(\theta _j|y)$ (orange dashed lines) obtained with the same priors, $\epsilon$, *T*, *w*, *M* and *N* as before, for different values of the time step $\varDelta$. In particular, we choose $\varDelta = 5\cdot 10^{-3}$ (top panels), $\varDelta =7.5 \cdot 10^{-3}$ (middle panels) and $\varDelta =10^{-2}$ (lower panels). The posteriors obtained from Algorithm 1 (ii) successfully targets $\pi _{\text {ABC}}(\theta |y)$, even for a time step as large as $\varDelta =10^{-2}$. On the contrary, Algorithm 1 (iii) is not even applicable. Indeed, the numerical scheme computationally pushes the amplitude of the oscillator towards infinity, resulting in a computer overflow, i.e. ${\widetilde{Y}}_\theta (t_i) \approx \infty$. Thus, neither ${\hat{f}}_{{{\tilde{y}}}_\theta }$ nor ${\hat{S}}_{{{\tilde{y}}}_\theta }$ can be computed and the density $\pi _{\text {ABC}}^e(\theta |y)$ cannot be derived.

**Fig. 5:**
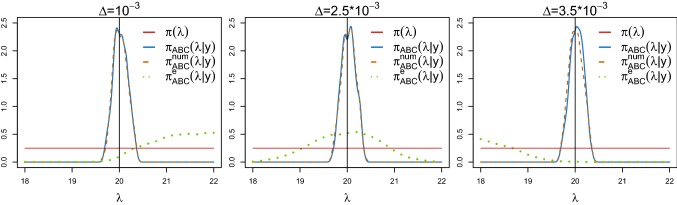
ABC posterior densities of $\theta =\lambda$ of the weakly damped stochastic oscillator (23) obtained from Algorithm 1 (i) using the exact simulation scheme (14) (blue solid lines), (ii) using the splitting scheme (22) (orange dashed lines) and (iii) using the Euler–Maruyama method (9) (green dotted lines) for different choices of the time step $\varDelta$. The horizontal red lines and the vertical black lines represent the uniform priors and the true parameter values, respectively. (Color figure online)

As a further illustration of the poor performance of the Euler–Maruyama scheme, even for smaller choices of $\varDelta$, we now consider the simplest possible scenario where we only estimate one parameter, namely $\theta =\lambda$. We set $N=10^5$, $M=10$, $\epsilon =1{\text {st}}$ percentile and we choose a uniform prior $\lambda \sim U(10,30)$. To be able to derive $\pi _{\text {ABC}}^{e}(\lambda |y)$, we simulate the synthetic data using the Euler–Maruyama method with the time steps $\varDelta =10^{-3}$, $2.5 \cdot 10^{-3}$ and $3.5\cdot 10^{-3}$. Figure 5 shows the three ABC posterior densities $\pi _{\text {ABC}}(\theta |y)$ (blue solid lines), $\pi _{\text {ABC}}^{\text {num}}(\theta |y)$ (orange dashed lines) and $\pi _{\text {ABC}}^{e}(\theta |y)$ (green dotted lines) for the different choices of $\varDelta$. The horizontal red lines and the black vertical lines denote the uniform prior and the true parameter value, respectively. In all cases, Algorithm 1 (iii) does not lead to a successful inference. In addition, these results are not stable for the different choices of $\varDelta$, and the derived ABC posterior density may not even cover the true parameter value.

## Validation of the spectral density-based and measure-preserving ABC Algorithm 1 (ii) on simulated and real data

We now illustrate the performance of Algorithm 1 (ii) by applying it to the stochastic JR-NMM. We rely on the efficient numerical splitting scheme (17) to guarantee measure-preserving synthetic data generation within the ABC framework. After estimating the parameters from simulated data, we infer them from real EEG data. In the available supplementary material, we illustrate the performance of Algorithm 1 (ii) also on the nonlinear damped stochastic oscillator, an extended version of the weakly damped harmonic oscillator discussed in Sect. 4.

### The stochastic Jansen and Rit neural mass model

The stochastic JR-NMM describes the electrical activity of an entire population of neurons through their average properties by modelling the interaction of the main pyramidal cells with the surrounding excitatory and inhibitory interneurons. The model has been reported to successfully reproduce EEG data and is applied in the research of neurological disorders such as epilepsy or schizophrenia (Wendling et al. 2000, 2002). The model is a 6-dimensional SDE of the form

25 $$\begin{aligned} \begin{aligned}&\hbox {d} \begin{pmatrix} Q(t) \\ P(t) \end{pmatrix} = \begin{pmatrix} 0 \\ \varSigma _\theta \end{pmatrix} \hbox {d}W(t) \\&+\,\begin{pmatrix} P(t) \\ -\varGamma ^2Q(t) -2\varGamma P(t) + G(Q(t);\theta ) \end{pmatrix} \hbox {d}t, \\ \end{aligned} \end{aligned}$$

where the 6-dimensional solution process is given by $\mathbf{X }=(\mathbf{Q },\mathbf{P })^{\prime }$ with components ${\mathbf {Q}}=({\mathbf {X}}_{{\mathbf {1}}},{\mathbf {X}}_{{\mathbf {2}}}, {\mathbf {X}}_{{\mathbf {3}}})^{\prime }$ and ${\mathbf {P}}=({\mathbf {X}}_{{\mathbf {4}}},{\mathbf {X}}_{{\mathbf {5}}},{\mathbf {X}}_{{\mathbf {6}}})^{\prime }$. None of the coordinates of $\mathbf{X }$ is directly observed. Only the difference between the second and third coordinates can be measured with EEG recording techniques, yielding the output process

$$\begin{aligned} \mathbf{Y }_\theta ={\mathbf {X}}_{{\mathbf {2}}}-{\mathbf {X}}_{{\mathbf {3}}}. \end{aligned}$$

In (25), the diagonal diffusion matrix is given by $\varSigma _\theta =\hbox {diag}[\sigma _4, \sigma _5, \sigma _6] \in {\mathbb {R}}^{3\times 3}$ with $\sigma _i>0$, $i=4,5,6$. The matrix $\varGamma =\hbox {diag}[a,a,b] \in {\mathbb {R}}^{3\times 3}$ is also diagonal with coefficients $a,b>0$, representing the time constants of the excitatory and inhibitory postsynaptic potentials, respectively. The nonlinear displacement term is given by

$$\begin{aligned} G({\mathbf {Q}};\theta )= \begin{pmatrix} Aa[\hbox {Sigm}({\mathbf {X}}_{{\mathbf {2}}}-{\mathbf {X}}_{{\mathbf {3}}})] \\ Aa[\mu +C_2\hbox {Sigm}(C_1{\mathbf {X}}_{{\mathbf {1}}})] \\ Bb[C_4\hbox {Sigm}(C_3{\mathbf {X}}_{{\mathbf {1}}})] \end{pmatrix}, \end{aligned}$$

where the sigmoid function Sigm: ${\mathbb {R}} \rightarrow [0, v_{{\mathrm{max}}}]$ is defined as

$$\begin{aligned} \text {Sigm}(x):=\frac{v_{{\mathrm{max}}}}{1+\text {exp}[r(v_0-x)]}, \end{aligned}$$

with $$v_{{\mathrm{max}}}>0$$ referring to the maximum firing rate of the neural populations, $$v_0 \in {\mathbb {R}}$$ describing the value for which $$50 \ \%$$ of the maximum firing rate is attained and $$r>0$$ denoting the slope of the sigmoid function at $$v_0$$. The parameters entering in *G* are $$\mu $$, *A*, *B* and $$C_i$$, $$i=1,2,3,4$$$$\in {\mathbb {R}}^+$$. The coefficients *A* and *B* describe the average excitatory and inhibitory synaptic gain, respectively. The parameters $$C_i$$ are internal connectivity constants, which reduce to only one parameter *C*, by using the relations $$C_1=C$$, $$C_2=0.8C$$, $$C_3=0.25C$$ and $$C_4=0.25C$$; see Jansen and Rit (1995).

### Parameter inference from simulated data

Not all model parameters of the JR-NMM are of biological interest or can be simultaneously identified. For example, the noise coefficients $\sigma _4$ and $\sigma _6$ were introduced mainly for mathematical convenience in Ableidinger et al. (2017). To guarantee the existence of a unique invariant measure $\eta _{\mathbf{X }}$ on $({\mathbb {R}}^6,{\mathcal {B}}({\mathbb {R}}^6))$, they are required to be strictly positive. However, from a modelling point of view, only the parameter $\sigma :=\sigma _5$ plays a role. Hence, we fix $\sigma _4=0.01$ and $\sigma _6=1$. The coefficients *A*, *B*, *a*, *b*, $v_0$, $v_{{\mathrm{max}}}$ and *r* have been experimentally recorded; see, e.g. Jansen et al. (1993), Jansen and Rit (1995) and van Rotterdam et al. (1982). Thus, we fix them according to these values reported, for example, in Table 1 of Ableidinger et al. (2017). In contrast, the connectivity parameter *C*, which represents the average number of synapses between the neural subpopulations and controls to what extent the main population interacts with the interneurons, varies under different physiological constraints. Changing *C* allows, for example, a transition from $\alpha$-rhythmic activity to epileptic spiking behaviour; see, e.g. Ableidinger et al. (2017). Here, we focus on the $\alpha$-rhythmic activity, which has a frequency of around 10 Hz. Since the parameters $\sigma$ and $\mu$ are new in the SDE version (25), they have not yet been estimated. They can be interpreted as stochastic and deterministic external inputs coming from neighbouring or more distant cortical columns, respectively. Thus, together with the internal connectivity parameter *C*, they are of specific interest. Before inferring $\theta =(\sigma ,\mu ,C)$, we take into account the coefficients *A* and *B* to discuss a model-specific issue of identifiability.

#### Identifiability issues: the detection of an invariant manifold, i.e. a set of parameters yielding the same type of data

For the original JR-NMM, it has been shown that different combinations of the parameters *A*, *B* and *C* yield the same type of output, namely $\alpha$-rhythmic brain activity. Applying the proposed spectral density-based and measure-preserving ABC Algorithm 1 (ii) for the inference of $\theta =(A,B,C)$, with given $\mu =220$ and $\sigma =2000$, we confirm that the same nonidentifiability arises for the SDE version (25). We choose $M=30$ observed paths generated assuming

$$\begin{aligned} \theta ^t=(A^t,B^t,C^t)=(3.25,22,135), \end{aligned}$$

as suggested in the literature (Jansen and Rit 1995). The reference data and the synthetic data are generated over a time interval of length $T=200$ and using a time step $\varDelta =2 \cdot 10^{-3}$. Within the algorithm, we generate $N=2.5 \cdot 10^6$ synthetic datasets. We choose the weight *w* in (7) according to the procedure introduced in Sect. 2.2 (based on $L=10^5$ iterations) and fix the tolerance level $\epsilon =0.04{\text {th}}$ percentile to keep $10^3$ of all the sampled values for $\theta$, as in the previous examples. Further, we choose independent uniform prior distributions, namely

$$\begin{aligned} A \sim {\mathcal {U}}(1,10), \qquad B \sim {\mathcal {U}}(10,100), \qquad C \sim {\mathcal {U}}(10,600). \end{aligned}$$

**Fig. 6:**
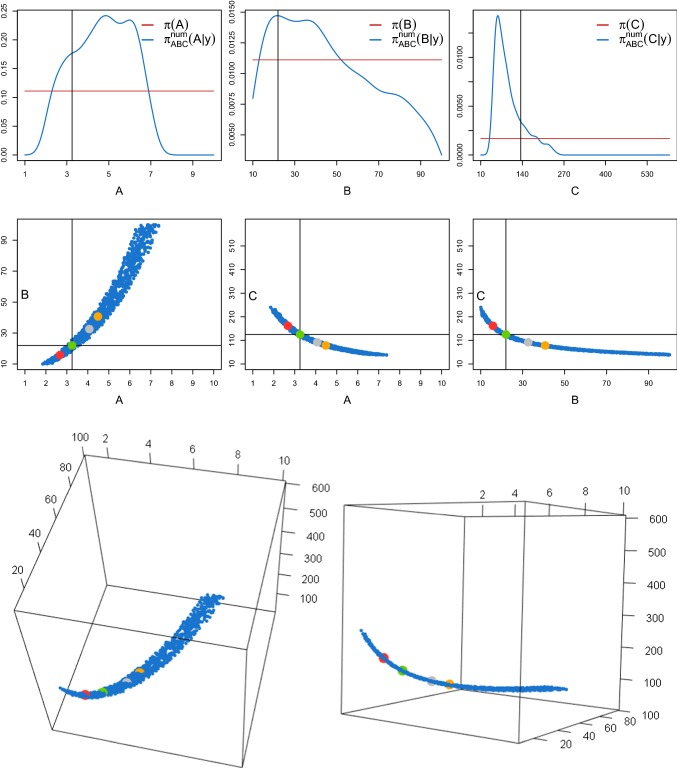
Top panels: ABC marginal posterior densities $\pi _{\text {ABC}}^{\text {num}}(\theta _j|y)$ (blue lines) of $\theta =(A,B,C)$ of the stochastic JR-NMM (25) obtained from Algorithm 1 (ii). The horizontal red lines and the vertical black lines represent the uniform priors and the true parameter values, respectively. Middle panels: Pairwise scatterplots of the kept ABC posterior samples. Lower panels: Two different views of a 3-dimensional scatterplot of the kept ABC posterior samples within a cuboid formed by the prior. The green dot corresponds to $\theta ^{t}$, and the red, orange and grey dots represent highlighted samples from the ABC posterior lying on the invariant manifold. (Color figure online)

**Fig. 7:**
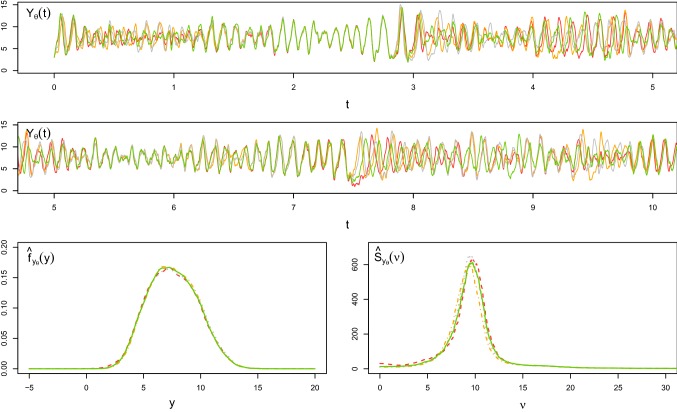
Top and middle panel: Four paths of the output process $\mathbf{Y }_\theta ={\mathbf {X}}_{{\mathbf {2}}}-{\mathbf {X}}_{{\mathbf {3}}}$ of the stochastic JR-NMM (25) generated under $\theta ^t$ (green lines) and with the three highlighted kept ABC posterior samples lying on the invariant manifold of Fig. 6 (red, orange and grey lines) using the same pseudo-random numbers. Lower panels: Corresponding estimated invariant densities (left) and estimated spectral densities (right). (Color figure online)

Figure 6 (top panels) shows the marginal ABC posterior densities $\pi _{\text {ABC}}^{\text {num}}(\theta _j|y)$ and the uniform prior densities $\pi (\theta _j)$. Clearly, the parameters cannot be inferred simultaneously. The kept ABC posterior values of the parameters *A*, *B* and *C* are strongly correlated, as observed in the pairwise scatterplots (middle panels) and in the 3-dimensional scatterplot (two different views, lower panels). The cuboid covers all possible values for $\theta$ drawn from the prior. After running the ABC algorithm, the kept values of $\theta$ from the ABC posterior form an invariant manifold, in the sense that all the parameter values $\theta$ lying on this manifold yield similar paths ${\tilde{y}}_\theta$ of the output process. This is shown in Fig. 7, where we report four trajectories that have been simulated with the same pseudo-random numbers but using the parameters $\theta ^t$ (green dot in Fig. 6) and three of the kept ABC posterior samples lying on the invariant manifold (red, orange and grey dots in Fig. 6). A segment of $T=10$ is split in the top and middle panels. In addition, we visualise the corresponding estimated invariant densities (bottom left) and invariant spectral densities (bottom right). This explains why the parameters *A*, *B* and *C* are not simultaneously identifiable from the observed data. Similar results are obtained when choosing smaller values of $\epsilon$. Interestingly, when increasing $\epsilon$, a second invariant manifold arises. Values for $\theta$ lying on this manifold yield similar estimated densities and spectral densities that slightly deviate from those derived under the observed data (cf. Section 3 of the supplementary material).

**Fig. 8:**
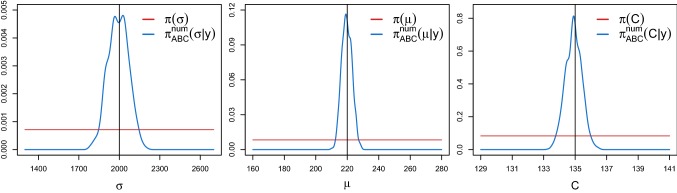
ABC marginal posterior densities $\pi _{\text {ABC}}^{\text {num}}(\theta _j|y)$ (blue lines) of $\theta =(\sigma ,\mu ,C)$ of the stochastic JR-NMM (25) obtained from Algorithm 1 (ii). The horizontal red lines and the vertical black lines represent the uniform priors and the true parameter values, respectively. (Color figure online)

Since the internal connectivity parameter *C* has an important neuronal meaning, in the following we assume *A* and *B* to be known and infer $\theta =(\sigma ,\mu ,C)$. The estimation of $\theta =(\sigma ,\mu )$ when *C* is known is reported in the supplementary material.

#### Inference of $\theta =(\sigma ,\mu ,C)$

Now, we keep the same ABC setting as before, except for defining $\epsilon =0.05{\text {th}}$ percentile. Further, we choose independent uniform priors $\pi (\theta _j)$ according to

$$\begin{aligned} \sigma \sim {\mathcal {U}}(1300,2700), \qquad \mu \sim {\mathcal {U}}(160,280), \qquad C \sim {\mathcal {U}}(129,141). \end{aligned}$$

The reference data are simulated under

$$\begin{aligned} \theta ^t=(\sigma ^t,\mu ^t,C^t)=(2000,220,135). \end{aligned}$$

In Fig. 8, we report the marginal ABC posterior densities $\pi _{\text {ABC}}^{\text {num}}(\theta _j|y)$ (blue lines), the uniform prior densities $\pi (\theta _j)$ (red lines) and the true parameter values $\theta ^t$ (black vertical lines). We obtain unimodal posterior densities, centred around the true parameter values. The posterior density of $\sigma$ is slightly broader compared to that obtained when *C* is known (cf. Figure 21 of the supplementary material). This results from a weak correlation that we detect among the kept ABC posterior samples of the parameters $\sigma$ and *C* (figures not reported). The posterior means are equal to

$$\begin{aligned} ({\hat{\sigma }}_{\text {ABC}}^{\text {num}},{\hat{\mu }}_{ABC}^{\text {num}},{\hat{C}}_{\text {ABC}}^{\text {num}})=(1992.253,219.744,134.899) \end{aligned}$$

and are thus close to $\theta ^t$. These results suggest an excellent performance of the proposed spectral density-based and measure-preserving ABC Algorithm 1 (ii).

**Fig. 9:**
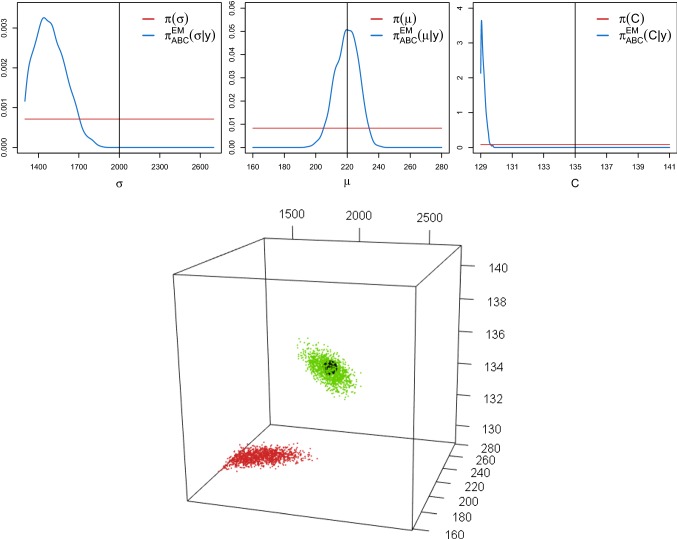
Top panels: Marginal ABC posterior densities $\pi _{\text {ABC}}^e(\theta _j|y)$ (blue lines) of $\theta =(\sigma ,\mu ,C)$ of the stochastic JR-NMM (25) obtained from Algorithm 1 (iii) using the non-preservative Euler–Maruyama scheme (9). The horizontal red lines and the vertical black lines represent the uniform priors and the true parameter values, respectively. Lower panel: 3-dimensional scatterplot of the kept ABC posterior samples using Algorithm 1 (ii) (green dots; see the previous results reported in Fig. 8) and Algorithm 1 (iii) (red dots). The cuboid is formed by the prior. The black dot corresponds to $\theta ^{t}$. (Color figure online)

Similar satisfactory results are obtained even when adding a fourth parameter, for example, when inferring $\theta =(\sigma ,\mu ,C,b)$ (cf. Figure 22 of the supplementary material). When applying Algorithm 1 (ii) to real EEG data (cf. Figure 23 of the supplementary material), the marginal posterior for *b* is centred around the value $b=50$, which is that reported in the literature. Due to the existence of underlying invariant manifolds, identifiability issues, similar to those reported in Fig. 6, arise when adding further or other coefficients, revealing model-specific issues for the stochastic JR-NMM.

To illustrate again the importance of the structure preservation within the ABC method, we now apply Algorithm 1 (iii). We use the same conditions as before, except for a smaller time step $\varDelta =10^{-4}$ used for the generation of the observed reference data with the Euler–Maruyama method aiming for a realistic data structure. In Fig.9, we report the marginal ABC posterior densities $\pi _{\text {ABC}}^e(\theta _j|y)$ (top panels) and the uniform prior densities. In the 3-dimensional scatterplot of Fig. 9 (lower panel), the green dots in the middle of the cuboid represent the kept ABC posterior samples when applying Algorithm 1 (ii) (see the previous results reported in Fig. 8), which are nicely spread out around the true parameter vector $\theta ^t$ (black dot). The red dots correspond to the kept ABC posterior samples from $\pi _{\text {ABC}}^e(\theta |y)$. Hence, Algorithm 1 (iii) based on the Euler–Maruyama scheme provides a posterior that is far off from the true parameter vector.

**Fig. 10:**
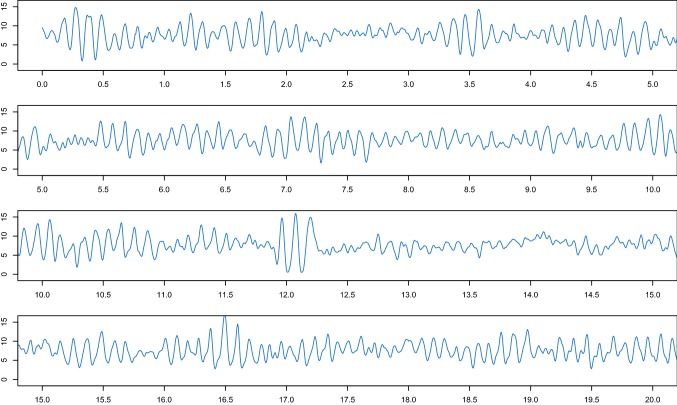
Visualisation of the first 20 seconds of one of the used $$\alpha $$-rhythmic EEG segments recorded with a sampling rate of 173.61 Hz, i.e. $$\varDelta \approx 5.76$$$$\text {ms}$$

### Parameter inference from real EEG data

Finally, we use the spectral density-based and measure-preserving ABC Algorithm 1 (ii) to estimate the parameter vector $$\theta =(\sigma ,\mu ,C)$$ of the stochastic JR-NMM from real EEG recordings. We use $$M=3$$$$\alpha $$-rhythmic recordings, rescaled to a realistic range. The EEG data were sampled according to a sampling rate of 173.61 Hz, i.e. a time step $$\varDelta $$ of approximately $$5.76\ \text {ms}$$ over a time interval of length $$T=23.6$$$$\text {s}$$. All measurements were carried out with a standardised electrode placement scheme; see Andrzejak et al. (2001) for further information on the data.^1^ Figure 10 shows the first 20 seconds of one of the observed EEG datasets. Here, we simulate $$N=5 \cdot 10^6$$ synthetic paths from the output process of the stochastic JR-NMM (25) over the same time interval *T* as the real data, with a time step $$\varDelta =2 \cdot 10^{-3}$$ and $$\epsilon =0.02{\text {nd}}$$ percentile. We choose independent uniform priors $$\pi (\theta _j)$$ according to

$$\begin{aligned} \sigma \sim {\mathcal {U}}(500,3500), \qquad \mu \sim {\mathcal {U}}(70,370), \qquad C \sim {\mathcal {U}}(120,150). \end{aligned}$$

**Fig. 11:**
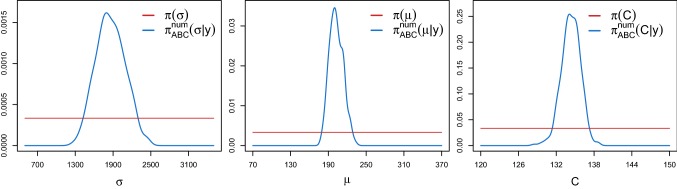
Marginal ABC posterior densities $\pi _{\text {ABC}}^{\text {num}}(\theta _j|y)$ (blue lines) of $\theta =(\sigma ,\mu ,C)$ of the stochastic JR-NMM (25) fitted on real EEG data using Algorithm 1 (ii). The red lines correspond to the uniform priors. (Color figure online)

Figure 11 shows the resulting marginal ABC posterior densities $\pi _{\text {ABC}}^\text {num}(\theta _j|y)$ and the uniform prior densities $\pi (\theta _j)$. All ABC marginal posteriors are unimodal, with means given by

$$\begin{aligned} ({\hat{\sigma }}_{\text {ABC}}^\text {num},{\hat{\mu }}_{ABC}^\text {num},{\hat{C}}_{\text {ABC}}^\text {num})=(1859.211,202.547,134.263). \end{aligned}$$

Since $\mu$ and $\sigma$ have not been estimated before, we cannot compare the obtained results with those available in the literature. The ABC posterior density for *C* is centred around $C=135$ that is the reference literature value for $\alpha$-rhythmic EEG data.

**Fig. 12:**
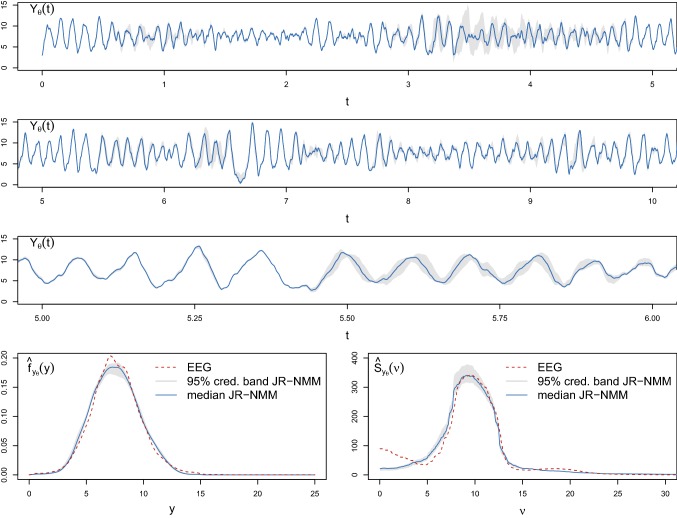
Top and middle top panel: Median (blue solid line) and 95% credible bands (shaded grey areas) of 10 *s* of trajectories of the output process of the stochastic JR-NMM (25) generated with the numerical splitting scheme (17) for $\varDelta =2 \cdot 10^{-3}$ and $T=23.6$ using the kept ABC posterior samples derived from Algorithm 1 (ii) under the same seed for pseudo-random numbers. Middle low panel: zoom of 1 *s* highlighting a frequency of around 10 Hz, confirming the $\alpha$-rhythmic behaviour. Lower panel: Estimated invariant density (left) and invariant spectral density (right) obtained from the EEG dataset shown in Fig. 10 (red dashed lines) plotted against the median (blue solid lines) and the 95% credible bands from the posterior predictive samples (shaded grey areas). (Color figure online)

In Fig. 12 (top two panels), we report the median (blue solid line) and the shaded 95% credible bands (obtained from the central posterior intervals) of the first 10 seconds of trajectories simulated from the fitted stochastic JR-NMM (25). The paths are generated with the numerical splitting scheme (17) for $\varDelta =2 \cdot 10^{-3}$ and $T=23.6$, using the $10^3$ kept ABC posterior samples derived from Algorithm 1 (ii) under the same seed for pseudo-random numbers. The narrow 95% confidence bands suggest how the kept ABC posterior samples yield similar paths. When using different pseudo-random numbers, both the median and the 95% credible bands of the generated trajectories look constant in time, as expected due to the underlying invariant distribution of the model (figures not shown). The bands show a similar oscillatory behaviour as shown in Fig. 10, with an approximate frequency of 10 Hz, successfully reproducing the underlying $\alpha$-rhythmic behaviour. This can be clearly distinguished in the middle lower panel, where we report a zoom of 1 second. The successful ABC inference is also confirmed by noting the matches between the invariant densities (bottom left) and the invariant spectral densities (bottom right) estimated from the EEG recording shown in Fig. 10 (red dashed lines) and from the fitted model when considering the median (blue solid lines) and the 95% credible bands (grey shaded areas). The match is poor only for low frequencies of the invariant spectral density, even when choosing broader priors. This may result from a lack of fit of the JR-NMM or of stationarity in the considered EEG data. A deeper investigation of the model (including adapted versions, see, e.g. Wendling et al. 2002) and of its ability in reproducing real EEG data is currently under investigation, but it is out of the scope of this work.

## Conclusion

When performing parameter inference through ABC, crucial and non-trivial tasks are to propose suitable summary statistics and distances to compare the observed and the synthetic datasets. When the underlying models are stochastic, repeated simulations from the same parameter setting yield different outputs, making the comparison between the observed and the synthetic data more difficult. To derive summary statistics that are less sensitive to the intrinsic randomness of the stochastic model, we propose to map the data to their invariant density and invariant spectral density, estimated by a kernel density estimator and a smoothed periodogram, respectively. By doing this, different trajectories of the output process are mapped to the same objects only when they are generated from the same underlying parameters, provided that all parameters are simultaneously identifiable. These transformations are based on the existence of an underlying invariant measure for the model, fully characterised by the parameters. A necessary condition of ABC, and of all other simulation-based methods, is the ability to generate data from the model. This is often taken for granted but, in general, it is not the case. Indeed, exact simulation is rarely possible and property-preserving numerical methods have to be derived.

The combination of the measure-preserving numerical splitting schemes and the use of the spectral density-based distances in the ABC algorithm lead to a successful inference of the parameters, as illustrated on stochastic Hamiltonian type equations when the process $\mathbf{Y }_\theta$ is observed without measurement error. We validated the proposed ABC approach on both linear model problems, allowing for an exact simulation of the synthetic data, and nonlinear problems, including an application to real EEG data. Our choice of the crucial ingredients (summary statistics and distances based on the underlying invariant distribution and a measure-preserving numerical method) yields excellent results even when applied to ABC in its basic acceptance–rejection form. However, they can be directly applied to more advanced ABC algorithms. In contrast, the ABC method based on the Euler–Maruyama scheme drastically fails. Its performance may improve for “small enough” time steps. However, there is a trade-off between the runtime and the acceptance performance of Algorithm 1 (iii). Indeed, the simulation of one trajectory with a time step $10^{-4}$ requires approximately hundred times more than the generation of one trajectory using a time step $10^{-2}$. Hence, a runtime of a few hours would turn to months. In addition, even for “arbitrary small” time steps, one cannot guarantee that the Euler–Maruyama scheme preserves the underlying invariant measure. For these reasons, it is crucial to base our ABC method on the reliable measure-preserving numerical splitting scheme combined with the invariant measure-based distances. Our results were discussed in the case of an observable 1-dimensional output process. However, the approach can be directly applied to *d*-dimensional output processes, $d>1$, as long as the underlying SDEs are characterised by an invariant distribution and a measure-preserving numerical method can be derived. In particular, one can compute the distances in (8) for each of the *d* components and derive a global distance by combining them, e.g. via their sum. Moreover, to account for possible dependences between the observed components, one can incorporate the cross-spectral densities which are expected to provide further information resulting in an improvement in the performance of the method. An investigation in this direction is currently undergoing. Finally, our proposed ABC method may be also used to investigate invariant manifolds characterised by sets of parameters yielding the same type of data, as illustrated on the stochastic JR-NMM. This may result in a better understanding of the qualitative behaviour of the underlying model and its ability of reproducing the true features of the modelled phenomenon.

## Supplementary material

Further illustrations of the proposed ABC method are available in the provided supplementary material. In particular, we illustrate the performance of: (a) Algorithm 1 (i) for the estimation of the parameters of the critically damped stochastic oscillator, for which the exact simulation is possible; Algorithm 1 (i) applied to the critically and weakly damped stochastic harmonic oscillators (see Sect. 4) when estimating a smaller number of parameters; (b) Algorithm 1 (ii) for the estimation of the parameters of a nonlinear damped stochastic oscillator; (c) Algorithm 1 (ii) for the estimation of the two parameters $\theta =(\sigma ,\mu )$ of the stochastic JR-NMM, which are of specific interest; (d) Algorithm 1 (ii) for the estimation of $\theta =(\sigma ,\mu ,C,b)$ of the stochastic JR-NMM, based on simulated and real EEG data. Moreover, we provide an investigation of the influence of $\epsilon$ on the identifiability issues discussed in Sect. 5.2.1.

## Electronic supplementary material

Below is the link to the electronic supplementary material.

Supplementary material 1 (pdf 1592 KB)

## Footnotes

1 The data are available on: http://ntsa.upf.edu/downloads/andrzejak-rg-et-al-2001-indications-nonlinear-deterministic-and-finite-dimensional.
