# Supplementary material for: Spectral density-based and measure-preserving ABC for partially observed diffusion processes. An illustration on Hamiltonian SDEs
Source: Stat Comput. 2019 Nov 5;30(3):627–48. doi: 10.1007/s11222-019-09909-6 (PMC7026277; doi:10.1007/s11222-019-09909-6)
Supplement: Supplementary file 1 — Supplementary material 1 (pdf 1592 KB) [file 11222_2019_9909_MOESM1_ESM.pdf]

Supplementary Material

Spectral Density-Based and Measure-Preserving ABC for partially observed diffusion processes.

An illustration on Hamiltonian SDEs.

Statistics and Computing

Evelyn Buckwar
Evelyn.Buckwar@jku.at

Massimiliano Tamborrino
Massimiliano.Tamborrino@jku.at

Irene Tubikanec
Irene.Tubikanec@jku.at

Institute for Stochastics
Johannes Kepler University Linz
Altenberger Straße 69, 4040 Linz, Austria

Supplementary Material

In this supplementary material, we extend the illustration of the performance of the proposed ABC approach by more examples. In particular, we consider two additional SDEs. First, the critically damped harmonic oscillator (23), fulfilling $\lambda^2 - \gamma^2 = 0$, for which an exact simulation of sample paths is possible, allowing for a validation of Algorithm 1 (i). Second, a nonlinear weakly damped stochastic oscillator, for which we need to apply a measure-preserving numerical splitting scheme, and thus investigate Algorithm 1 (ii). We then provide an investigation of the influence of the tolerance level ϵ on the identifiability issues for the inference of the parameters $\theta = (A, B, C)$ in the stochastic JR-NMM (25) (related to Section 5.2.1 of the main manuscript). Moreover, we also report the simultaneous inference of the new parameters $\theta = (\sigma, \mu)$ in the stochastic JR-NMM (25), when the connectivity parameter C is known (while in the main manuscript, we estimate $\theta = (\sigma, \mu, C)$). Finally, we report the estimation of $\theta = (\sigma, \mu, C, b)$, based on both simulated and real EEG data.

1 Validation of the Spectral Density-Based ABC Algorithm 1 (i)

We denote by Model Problem 1 (MP1) the critically damped harmonic oscillator obtained from (23) with $\lambda^2 - \gamma^2 = 0$ (introduced below), and with Model Problem 2 (MP2) the weakly damped harmonic oscillator, satisfying $\lambda^2 - \gamma^2 > 0$ (see Section 4 of the main manuscript). Figure 13 shows two realisations of the output process of MP1 generated with the same choice of parameters. Figure 14 shows two paths of the output process of MP2 simulated under the same parameter setting. We perform a step by step investigation of Algorithm 1 (i), starting with the estimation of one single model parameter and closing with the successful inference of all parameters.

1.1 Critically damped stochastic harmonic oscillator: The model and its properties

We recall the damped harmonic oscillator (23), focusing on the critically damped case, i.e., $\lambda^2 - \gamma^2 = 0$. We assume that the 2-dimensional process $\mathbf{X} = (\mathbf{Q}, \mathbf{P})'$ is partially observed through the second component, i.e., $\mathbf{Y}_\theta = \mathbf{P}$. The invariant distribution $\eta_\mathbf{X}$ of the process $\mathbf{X}$ is given by

$$\eta_\mathbf{X} = \lim_{t\to\infty} \eta_\mathbf{X}(t) = \mathcal{N}\left(\begin{pmatrix} 0 \\ 0 \end{pmatrix}, \begin{pmatrix} \frac{\sigma^2}{4\gamma^3} & 0 \\ 0 & \frac{\sigma^2}{4\gamma} \end{pmatrix} \right).$$

Consequently, the invariant distribution $\eta_{\mathbf{Y}_\theta}$ of the output process $\mathbf{Y}_\theta$ equals

$$\eta_{\mathbf{Y}_\theta} = \mathcal{N}\left(0, \frac{\sigma^2}{4\gamma}\right),$$

and the autocovariance function is given by

$$r_\theta(\Delta) = \frac{\sigma^2}{4} e^{-\gamma\Delta} \left[\frac{1}{\gamma} - \Delta \right].$$

1.2 Task 1: Inferring one parameter

At first, we estimate one specific parameter θ of the model problems, keeping the others fixed. For MP1, we set $\theta = \gamma$ and fix $\sigma = 2$. For MP2, we focus on $\theta = \lambda$, fixing $\gamma = 1$ and $\sigma = 2$. Algorithm 1 (i) is applied to both model problems with $M = 10$ observed paths simulated with the exact scheme (14) using a time step $\Delta = 10^{-2}$ over a time interval of length $T = 10^3$. In addition, we generate $N = 10^5$ synthetic datasets over the same time domain with equal time steps using the exact simulation scheme (14). We set the tolerance level to $\epsilon = 1^{\text{st}}$ percentile of the calculated distances. Furthermore, we choose uniform prior distributions $\pi(\theta)$ according to

$$\theta = \begin{cases} \gamma \sim \mathcal{U}(0.1, 5) & \text{for MP1,} \\ \lambda \sim \mathcal{U}(10, 30) & \text{for MP2} \end{cases}.$$

We use the same parameter setting as in Figure 13 and Figure 14 for the simulation of the observed reference datasets. In particular, the true parameter values are

$$\theta^t = \begin{cases} \gamma^t = 1 & \text{for MP1,} \\ \lambda^t = 20 & \text{for MP2} \end{cases}.$$

In Figure 15, we report the results of the proposed Spectral Density-Based ABC Algorithm 1 (i). The left panel (referring to MP1) and the right panel (referring to MP2) show the ABC posterior densities $\pi_{\text{ABC}}(\theta|y)$ (blue lines). The horizontal red and vertical black lines denote the prior densities and the true parameter values, respectively. It is remarkable how the flat uniform prior densities are updated by means of the observed data resulting in narrow and unimodal posterior densities that are centered around the true parameter values. The ABC posterior means for this and the other scenarios (i.e. the inference of two and three parameters) are reported in Table 1.

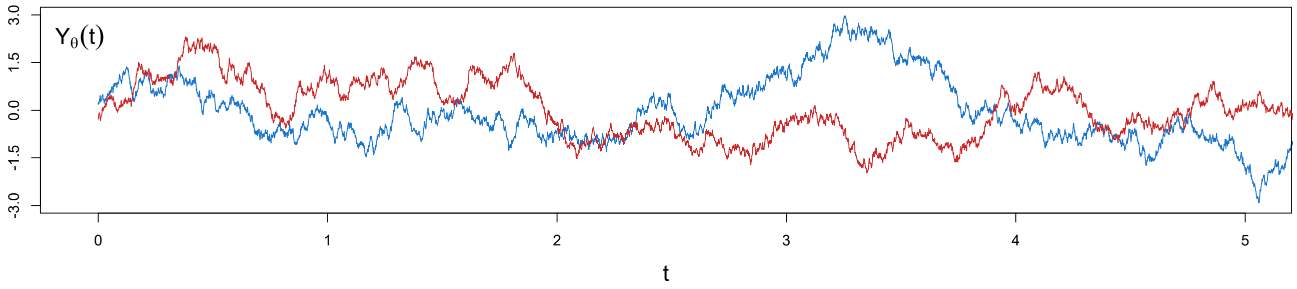

Fig. 13. Two paths of the output process $\mathbf{Y}_\theta = \mathbf{P}$ of the critically damped stochastic harmonic oscillator (MP1) for a noise intensity $\sigma^t = 2$ and parameter $\gamma^t = 1$

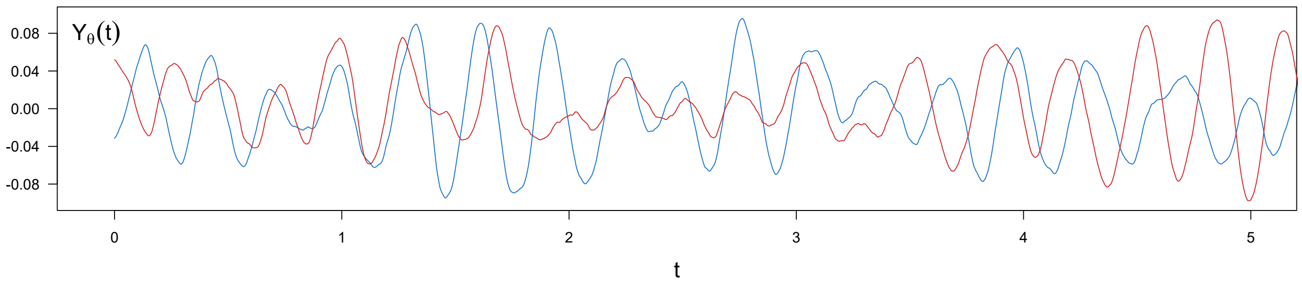

Fig. 14. Two paths of the output process $\mathbf{Y}_\theta = \mathbf{Q}$ of the weakly damped stochastic harmonic oscillator (MP2) for a noise intensity $\sigma^t = 2$, $\gamma^t = 1$ and a damping force $\lambda^t = 20$

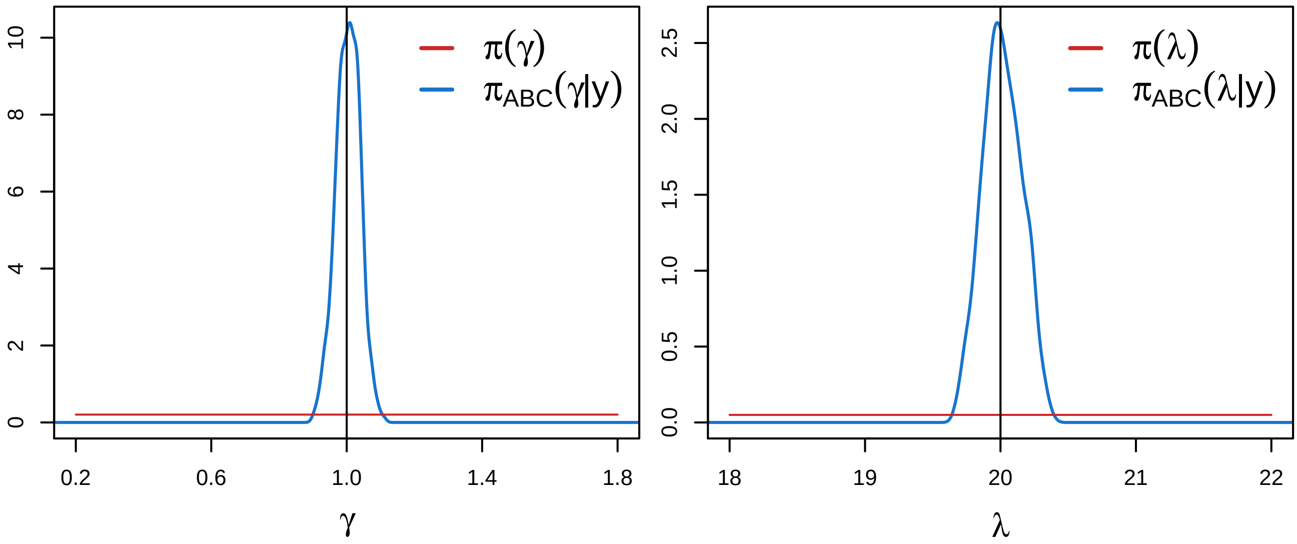

Fig. 15. ABC posterior densities $\pi_{\mathrm{ABC}}(\theta|y)$ (blue lines) of MP1 (left panel) and MP2 (right panel) obtained from Algorithm 1 (i). The horizontal red and vertical black lines denote the uniform priors (not shown according to the full domain) and the true parameter values, respectively

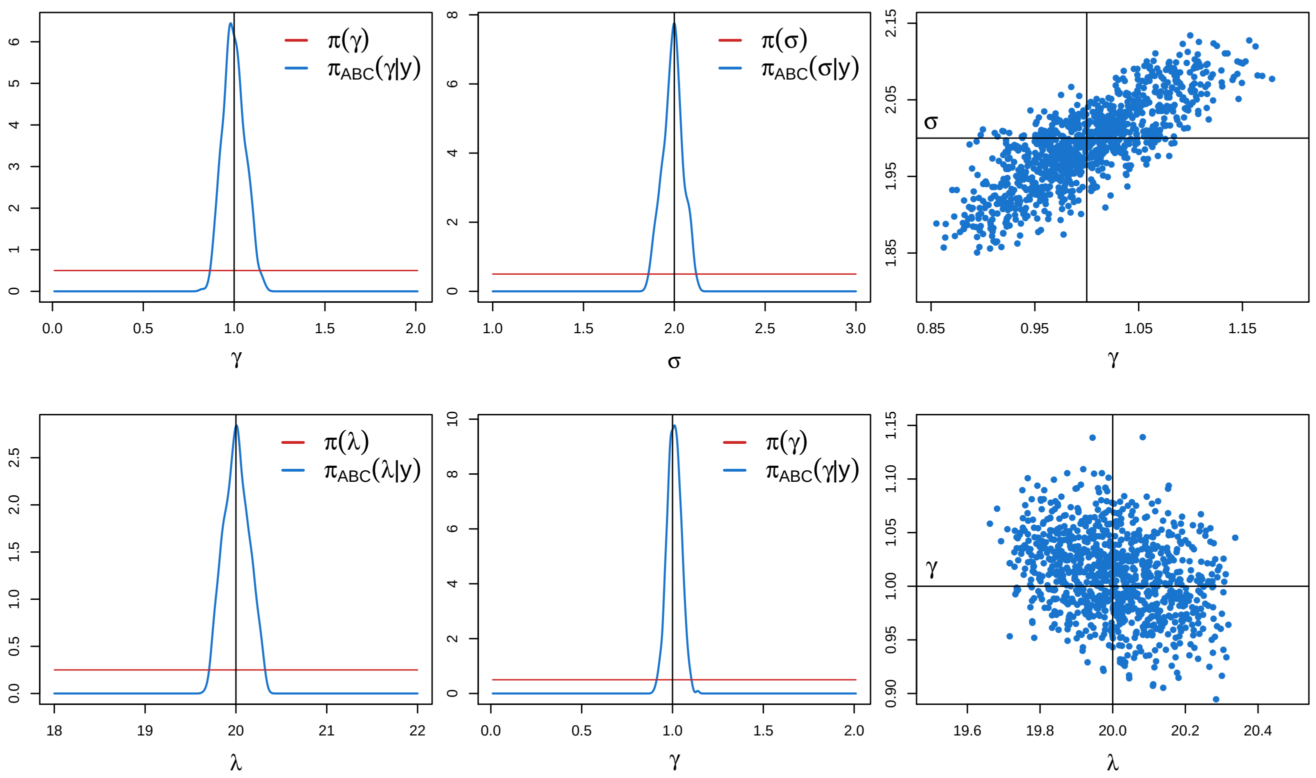

Fig. 16. ABC marginal posterior densities $\pi_{\text{ABC}}(\theta_j|y)$ (blue lines) of MP1 (left and middle top panels) and MP2 (left and middle lower panels) obtained from Algorithm 1 (i). The horizontal red and vertical black lines denote the uniform priors and the true parameter values, respectively. Scatterplots of the kept ABC posterior samples for MP1 and MP2 are reported in the right top and right lower panel, respectively

1.3 Task 2: Inferring two parameters

We aim for the simultaneous estimation of two parameters, keeping the parameter $\sigma = 2$ fixed in MP2. In particular, we consider $\theta = (\gamma, \sigma)$ for MP1 and $\theta = (\lambda, \gamma)$ for MP2. We apply Algorithm 1 (i) combined with the exact scheme (14) under the same values for M, Δ and T as before. Now, we generate $N = 5 \cdot 10^5$ synthetic datasets and fix $\epsilon = 0.2^{\text{nd}}$ percentile of the calculated distances, keeping the same number of ABC posterior samples as before. We choose the independent uniform priors $\pi(\theta_j)$ according to

$$\theta_j = \begin{cases} \gamma \sim \mathcal{U}(0.01, 2.01) \text{ and } \sigma \sim \mathcal{U}(1,3) & \text{for MP1,} \\ \lambda \sim \mathcal{U}(18, 22) \text{ and } \gamma \sim \mathcal{U}(0.01, 2.01) & \text{for MP2} \end{cases}.$$

The true parameter values are

$$\theta^t = \begin{cases} (\gamma^t, \sigma^t) = (1,2) & \text{for MP1,} \\ (\lambda^t, \gamma^t) = (20,1) & \text{for MP2} \end{cases}.$$

The ABC marginal posterior densities $\pi_{\text{ABC}}(\theta_j|y)$ (blue lines) are reported in the left and middle panels of Figure 16 for MP1 (top panels) and MP2 (lower panels), while the right panels of Figure 16 show the scatterplots of the kept ABC posterior samples. Also in this case, the posteriors are unimodal and centered around the true parameter values. Note that, the support of $\pi_{\text{ABC}}(\lambda|y)$ for MP2 is approximately the same as in Figure 15, suggesting that, in the case of inferring two parameters, the proposed ABC method is able to identify the same region for λ as in the case of estimating one parameter. The reason is that the kept ABC posterior samples of λ and γ are not correlated, as it can be observed in the right lower panel of Figure 16. On the contrary, the support of $\pi_{\text{ABC}}(\gamma|y)$ for MP1 is broader than in Figure 15, due to a correlation among the kept ABC posterior samples of γ and σ (cf. right top panel of Figure 16). In spite of this, the ABC marginal posterior density resembles that derived when estimating only one parameter (cf. left panel of Figure 15).

1.4 Task 3: Inferring three parameters

The last goal is the simultaneous inference of all the three parameters $\theta = (\lambda, \gamma, \sigma)$ of MP2.[1] In Figure 3 (top panels) we report the ABC marginal posterior densities (blue lines) and the prior densities (red lines). In the lower panels, we show the pairwise scatterplots of the kept ABC posterior samples. The kept posterior values of λ turned out to be not correlated with those of the other two parameters, yielding approximately the same support as in Figure 15 and Figure 16. Similar to MP1, the kept ABC posterior samples of γ and σ are correlated (cf. right lower panel of Figure 3), leading to a support for γ broader than that in Figure 16. The ABC marginal posterior densities shown in Figures 3, 15 and 16, and the results reported in Table 1 highlight the good performance of the proposed Spectral Density-Based ABC Algorithm 1 (i) under the optimal condition of exact, and thus measure-preservative data simulation from the underlying model.

Table 1: Parameters of interest, true parameter values and ABC posterior means

θ	θ^t	$\hat{\theta}_{\text{ABC}}$
MP1		
γ	1	1.004
(γ, σ)	$(1, 2)$	$(0.9995, 1.991)$
MP2		
λ	20	20.014
(λ, γ)	$(20, 1)$	$(20.005, 1.009)$
$(\lambda, \gamma, \sigma)$	$(20, 1, 2)$	$(20.015, 1.002, 2.011)$

2 Validation of the Spectral Density-Based and Measure-Preserving ABC Algorithm 1 (ii) on an extended version of MP2

We now consider an extended non-linear version of the previously studied Model Problem 2. Due to the non-linearity in the model, an exact simulation scheme is not available. Hence, we consider the measure-preserving numerical splitting scheme (17), and thus investigate the performance of Algorithm 1 (ii).

[1] Task 3 is already presented in Subsection 4.2 of the main manuscript. For completeness, we recall it here.

2.1 A non-linear weakly damped stochastic oscillator

We consider a stochastic oscillator that incorporates a high-amplitude sine wave represented by the non-linear displacement term $G(\mathbf{Q}) = -10^3 \sin(\mathbf{Q})$. In particular, we study the 2-dimensional SDE

$$d\begin{pmatrix} Q(t) \\ P(t) \end{pmatrix} = \begin{pmatrix} 0 \\ \sigma \end{pmatrix} dW(t) + \begin{pmatrix} P(t) \\ -\lambda^2 Q(t) - 2\gamma P(t) + G(Q(t)) \end{pmatrix} dt \tag{26}$$

with the strictly positive parameters $\theta = (\lambda, \gamma, \sigma)$. The condition $\lambda^2 - \gamma^2 > 0$ guarantees a weak damping. The 2-dimensional solution process $\mathbf{X} = (\mathbf{Q}, \mathbf{P})'$ is partially observed through the first coordinate, i.e., $\mathbf{Y}_\theta = \mathbf{Q}$. Figure 17 shows two realisations of the output process generated with the same choice of parameters.

2.2 Parameter inference from simulated data

We assume to observe $M = 30$ paths of the output process simulated with the measure-preserving numerical scheme (17) over a time interval of length $T = 10^3$ using a time step $\Delta = 10^{-2}$ and the same true parameter values as in Figure 17, i.e.,

$$\theta^t = (\lambda^t, \gamma^t, \sigma^t) = (20, 1, 2).$$

We then use the same T and Δ to generate $N = 2 \cdot 10^6$ synthetic datasets within ABC. We further choose the tolerance level $\epsilon = 0.05^{\text{th}}$ percentile of the calculated distances, set $w = 0$ in (7) and use independent uniform prior distributions

$$\lambda \sim \mathcal{U}(18, 22), \quad \gamma \sim \mathcal{U}(0.01, 2.01) \quad \text{and} \quad \sigma \sim \mathcal{U}(1, 3).$$

Figure 18 shows the ABC marginal posterior densities $\pi^{\text{num}}_{\text{ABC}}(\theta_j|y)$. They are unimodal, narrow and centered around the true parameter values. The ABC posterior means are given by

$$(\hat{\lambda}^{\text{num}}_{\text{ABC}}, \hat{\gamma}^{\text{num}}_{ABC}, \hat{\sigma}^{\text{num}}_{ABC}) = (20.015, 1.008, 2.0105).$$

In spite of the presence of the non-linear term G, the inference via Algorithm 1 (ii) yields results similar to those obtained for MP2 when applying Algorithm 1 (i) under the exact data generation.

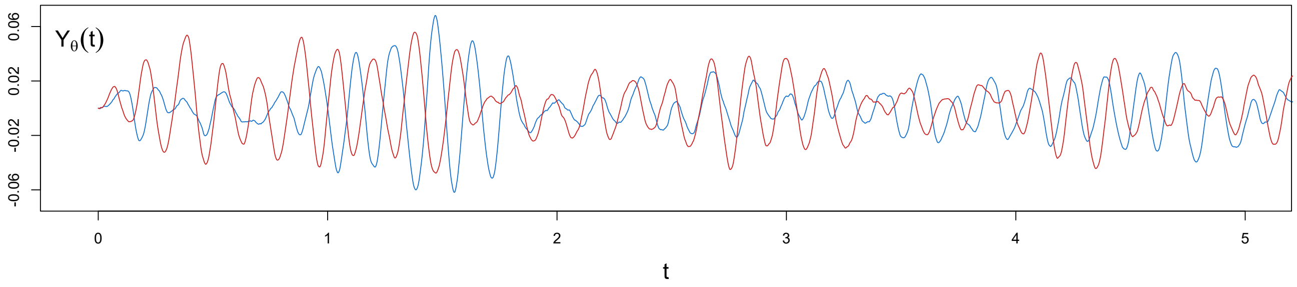

Fig. 17. Two paths of the output process $\mathbf{Y}_\theta = \mathbf{Q}$ of the non-linear oscillator (26) for $\theta^t = (\lambda^t, \gamma^t, \sigma^t) = (20, 1, 2)$

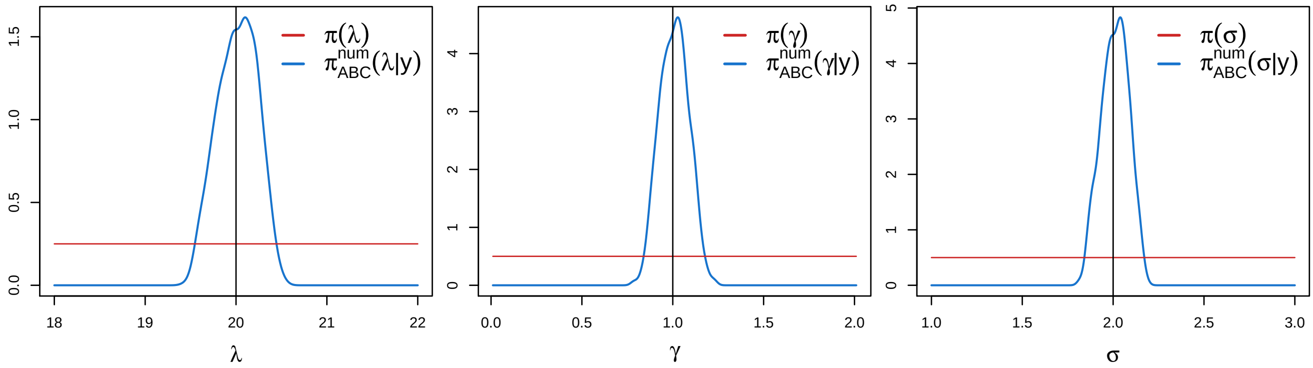

Fig. 18. ABC marginal posterior densities $\pi_{\text{ABC}}^{\text{num}}(\theta_j|y)$ (blue lines) of $\theta = (\lambda, \gamma, \sigma)$ of the non-linear weakly damped stochastic oscillator (26) obtained from Algorithm 1 (ii). The horizontal red and vertical black lines denote the uniform priors and the true parameter values, respectively

3 A note on the identifiability issues of the stochastic JR-NMM

We illustrate how a larger choice of the tolerance level ϵ influences the results presented in Section 5.2.1 of the main manuscript (see Figure 6 and Figure 7). Here we use the same ABC setting, except for choosing $\epsilon = 0.1^{\text{st}}$ percentile. Figure 19 (top panels) shows the marginal ABC posterior densities $\pi_{\text{ABC}}^{\text{num}}(\theta_j|y)$ and the marginal uniform prior densities. The kept ABC posterior samples are strongly correlated and form two distinct manifolds (middle and lower panels). We observe that values of θ lying on the second (the newly detected) manifold yield similar approximations of the density and spectral density that slightly deviate from those derived under θ^t. This is shown in Figure 20, where we report four trajectories (top and middle panel) that have been simulated with the same pseudo-random numbers under θ^t (green dot in Figure 19) and three of the kept ABC posterior samples lying on the second manifold (red, orange and grey dots in Figure 19) and the corresponding estimated invariant densities (bottom left) and spectral densities (bottom right). On one hand, this second invariant manifold suggests again an identifiability issue. On the other hand, it allows to discriminate between posterior samples yielding a better and poorer fit of the data, depending on whether the kept posterior samples lie in the first or the second manifold, respectively.

4 Application of the Spectral Density-Based and Measure-Preserving ABC Algorithm 1 (ii) for the inference of the new parameters $\boldsymbol{\theta} = (\boldsymbol{\sigma}, \boldsymbol{\mu})$ of the stochastic JR-NMM

We now infer $\theta = (\sigma, \mu)$ of the stochastic JR-NMM (25) (see Section 5 of the main manuscript). These are new parameters introduced by Ableidinger et al. (2017) in the SDE reformulation of the original JR-NMM (Jansen and Rit 1995). Differently from the other model parameters, these parameters have not yet been estimated in the literature. Here, we fix $C = 135$ and apply Algorithm 1 (ii) with $M = 30$, $N = 5 \cdot 10^5$, $\Delta = 2 \cdot 10^{-3}$ and $T = 200$. We fix $\epsilon = 0.2^{\text{nd}}$ percentile of the calculated distances and choose uniform prior distributions according to

$$\sigma \sim \mathcal{U}(1300, 2700) \quad \text{and} \quad \mu \sim \mathcal{U}(160, 280).$$

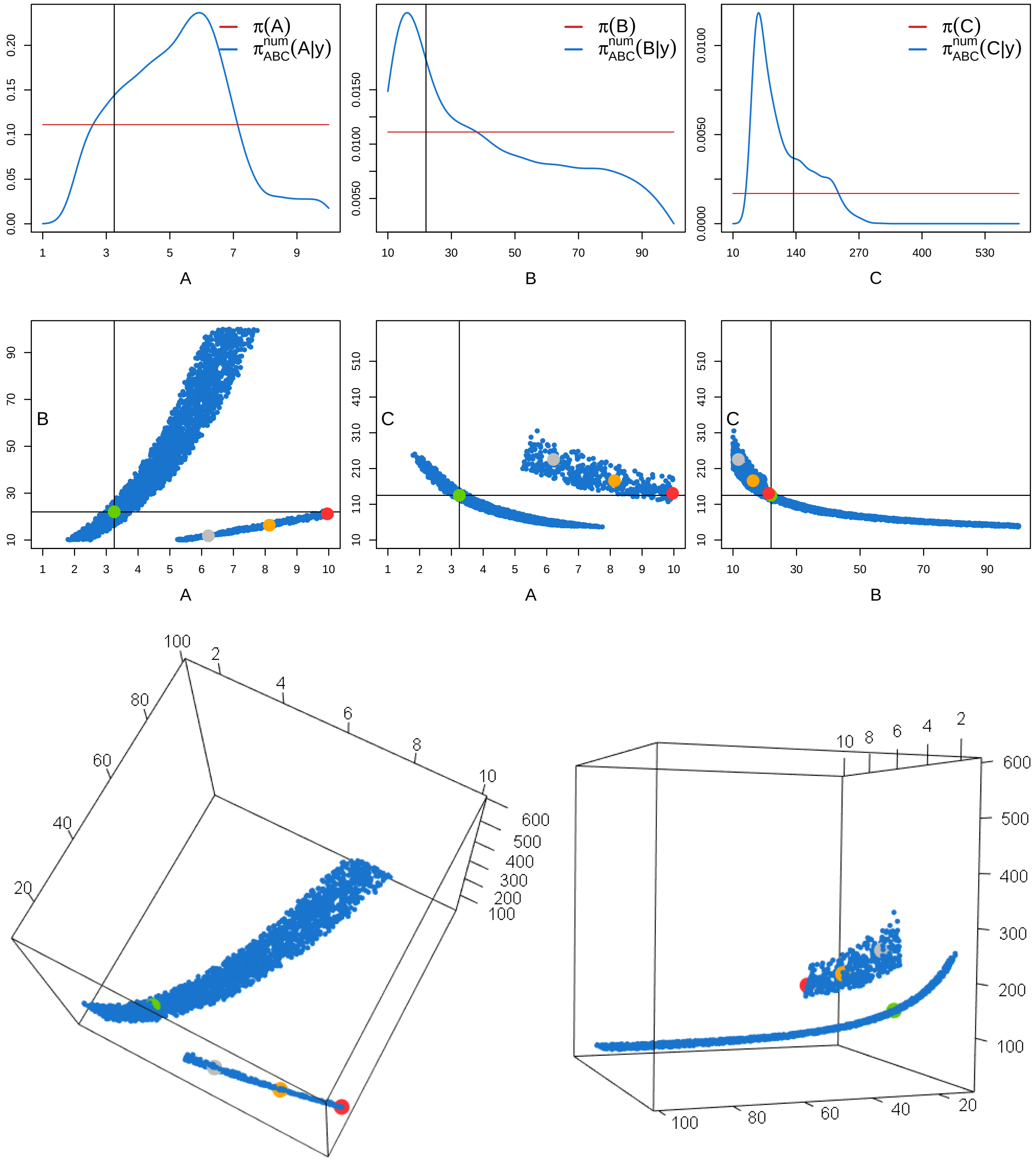

Fig. 19. Top panels: ABC marginal posterior densities $\pi^{\mathrm{num}}_{\mathrm{ABC}}(\theta_j|y)$ (blue lines) of $\theta = (A, B, C)$ of the stochastic JR-NMM (25) obtained from Algorithm 1 (ii). The horizontal red lines and the vertical black lines represent the uniform priors and the true parameter values, respectively. Middle panels: Pairwise scatterplots of the kept ABC posterior samples. Lower panels: Two different views of a 3-dimensional scatterplot of the kept ABC posterior samples within a cuboid formed by the prior. The green dot corresponds to θ^t and the red, orange and grey dots represent highlighted samples from the ABC posterior lying on the second invariant manifold

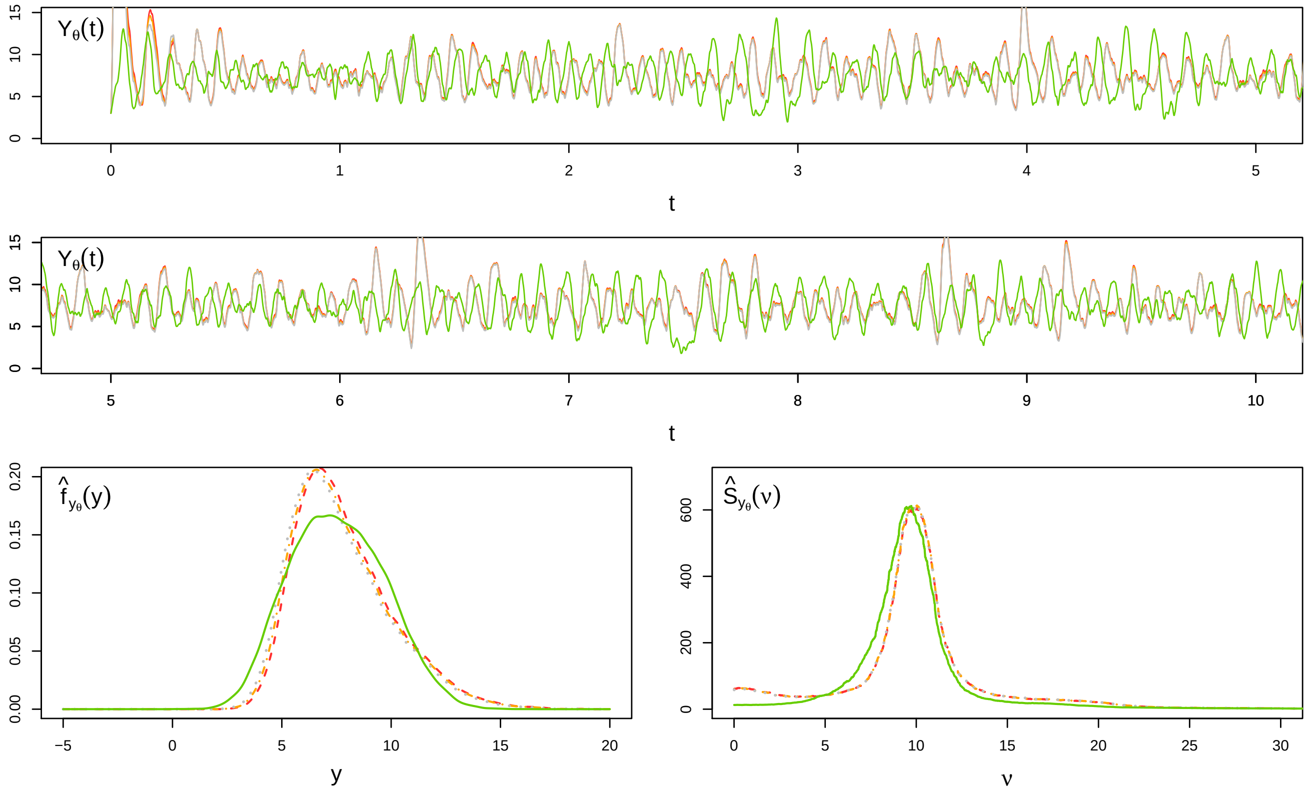

Fig. 20. Top and middle panel: Four paths of the output process $\mathbf{Y}_\theta = \mathbf{X_2} - \mathbf{X_3}$ of the stochastic JR-NMM (25) generated under θ^t (green lines) and with the three highlighted kept ABC posterior samples lying on the invariant manifold of Figure 19 (red, orange and grey lines) using the same random numbers. Lower panels: Corresponding estimated invariant densities (left) and estimated spectral densities (right)

The true parameter values used to generate the observed data are given by

$$\theta^t = (\sigma^t, \mu^t) = (2000, 220).$$

Figure 21 shows the ABC marginal posterior densities $\pi^{\text{num}}_{\text{ABC}}(\theta_j|y)$ (left and middle top panels) obtained by applying the Spectral Density-Based and Measure-Preserving ABC Algorithm 1 (ii). The posteriors are centered around the true parameter values, leading to marginal ABC posterior means given by

$$(\hat{\sigma}^{\text{num}}_{\text{ABC}}, \hat{\mu}^{\text{num}}_{\text{ABC}}) = (1985.936, 220.1364).$$

From the scatterplot of the kept ABC posterior samples of σ and μ (right top panel), we conclude that they are not correlated. The successful performance of the proposed ABC approach is also visible by looking at the contour plot of the ABC posterior density (lower panel). Indeed, the proposed algorithm is able to detect a plain region of posterior values for θ around θ^t.

5 Application of the Spectral Density-Based and Measure-Preserving ABC Algorithm 1 (ii) for the inference of $\boldsymbol{\theta} = (\boldsymbol{\sigma}, \boldsymbol{\mu}, \boldsymbol{C}, \boldsymbol{b})$ of the stochastic JR-NMM

We now demonstrate that we obtain satisfactory results even when inferring the parameters $\theta = (\sigma, \mu, C, b)$ of the stochastic JR-NMM (25). Since the parameters of main interest are σ, μ and C, in the main manuscript (see Section 5) we did not take into account the well-reported coefficient b, which takes the value $b = 50$ in the literature; see, e.g., Jansen and Rit (1995).

5.1 Inference from simulated data

We start with inferring $\theta = (\sigma, \mu, C, b)$ from simulated data and apply Algorithm 1 (ii) for $M = 30$, $N = 5{\cdot}10^6$, $\Delta = 2 \cdot 10^{-3}$ and $T = 200$. We fix $\epsilon = 0.004^{\text{th}}$ percentile and use the following uniform priors

$$\sigma \sim \mathcal{U}(1300, 2700), \quad \mu \sim \mathcal{U}(160, 280),$$

$$C \sim \mathcal{U}(129, 141) \quad \text{and} \quad b \sim \mathcal{U}(44, 56).$$

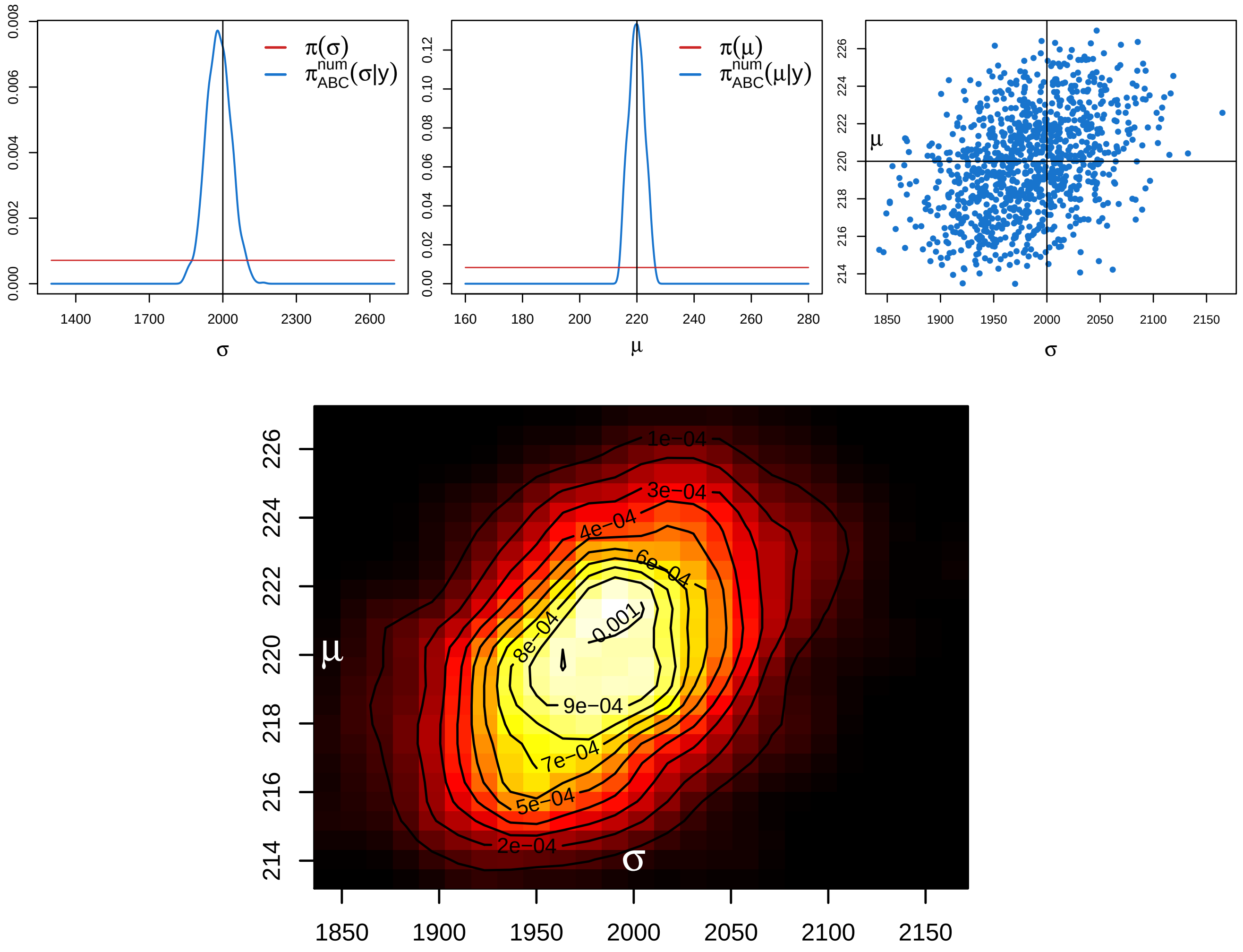

Fig. 21. ABC marginal posterior densities $\pi_{\text{ABC}}^{\text{num}}(\theta_j|y)$ (blue lines, left and middle top panels) of $\theta = (\sigma, \mu)$ of the stochastic JR-NMM (25) obtained from Algorithm 1 (ii). The horizontal red and vertical black lines denote the uniform priors and the true parameter values, respectively. Scatterplot of the kept ABC posterior samples (right top panel) and contour plot of the ABC posterior density (lower panel)

The reference data is generated under

$$\theta^t = (\sigma^t, \mu^t, C^t, b^t) = (2000, 220, 135, 50).$$

In Figure 22, we report the marginal ABC posterior densities $\pi_{\text{ABC}}^{\text{num}}(\theta_j|y)$, which are again centered around the true parameter values. The marginal posterior means are given by

$$(\hat{\sigma}_{\text{ABC}}^{\text{num}}, \hat{\mu}_{\text{ABC}}^{\text{num}}, \hat{C}_{\text{ABC}}^{\text{num}}, \hat{b}_{\text{ABC}}^{\text{num}}) = (1992.6, 219.7, 134.95, 50.05).$$

5.2 Inference from real EEG data

Finally, we infer $\theta = (\sigma, \mu, C, b)$ from real EEG data. Algorithm 1 (ii) is applied under the same conditions as in Subsection 5.3 of the main manuscript, except for fixing $\epsilon = 0.002^{\text{nd}}$ percentile of calculated distances and choosing the uniform priors according to

$$\sigma \sim \mathcal{U}(500, 3500), \quad \mu \sim \mathcal{U}(70, 370),$$

$$C \sim \mathcal{U}(120, 150) \quad \text{and} \quad b \sim \mathcal{U}(40, 60).$$

Figure 23 shows the unimodal marginal ABC posterior densities $\pi_{\text{ABC}}^{\text{num}}(\theta_j|y)$, yielding posterior means given by

$$(\hat{\sigma}_{\text{ABC}}^{\text{num}}, \hat{\mu}_{\text{ABC}}^{\text{num}}, \hat{C}_{\text{ABC}}^{\text{num}}, \hat{b}_{\text{ABC}}^{\text{num}}) = (1902.6, 200.2, 134.45, 50.46).$$

Focusing on the coefficient b, the corresponding marginal posterior density is centered around $b = 50$, which is the value reported in the literature.

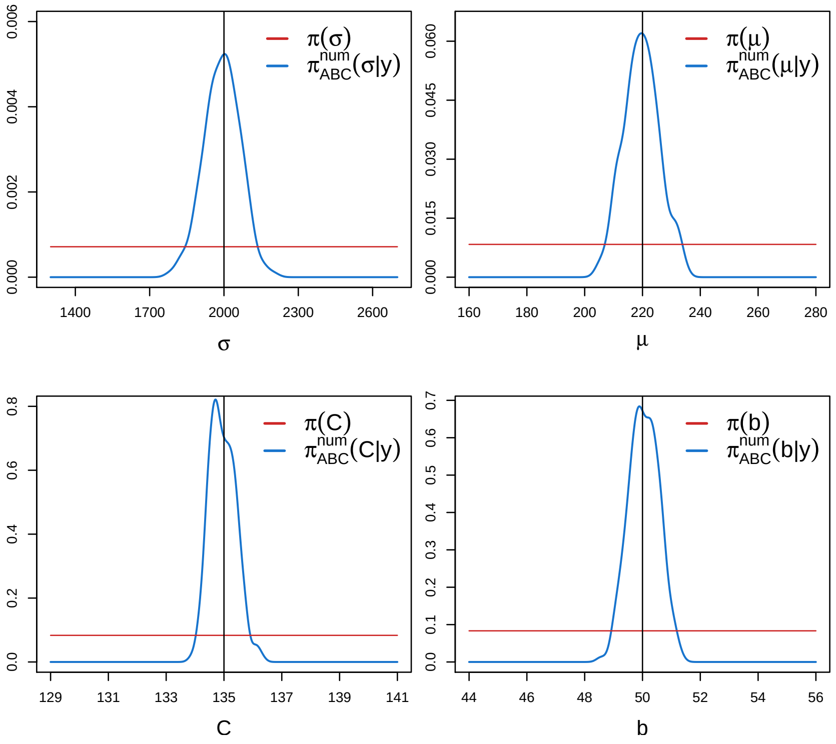

Fig. 22. ABC marginal posterior densities $\pi^{\text{num}}_{\text{ABC}}(\theta_j|y)$ (blue lines) of $\theta = (\sigma, \mu, C, b)$ of the stochastic JR-NMM (25) obtained from Algorithm (1) (ii). The horizontal red lines and the vertical black lines represent the uniform priors and the true parameter values, respectively

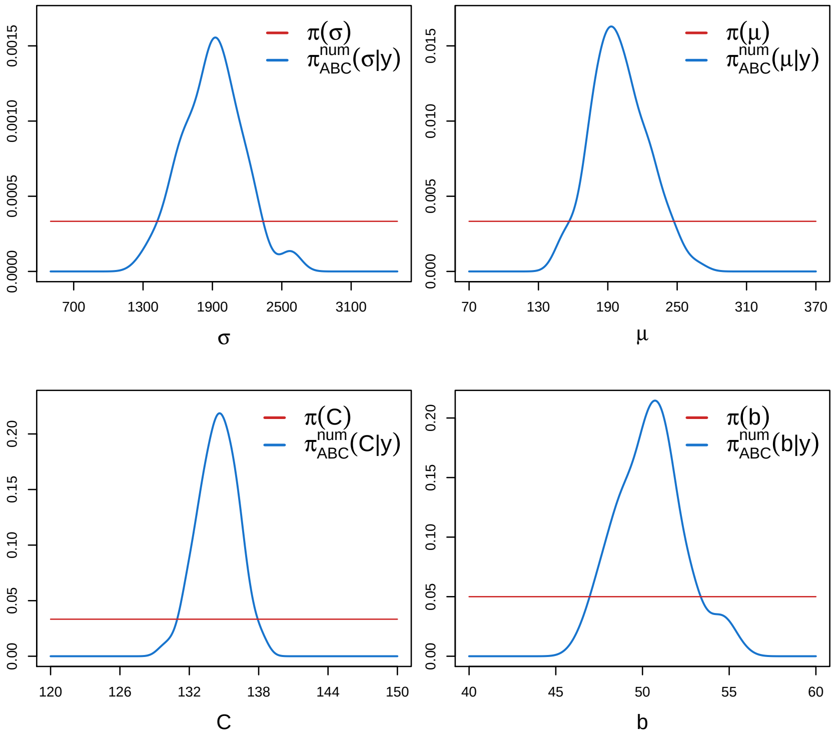

Fig. 23. Marginal ABC posterior densities $\pi^{\text{num}}_{\text{ABC}}(\theta_j|y)$ (blue lines) of $\theta = (\sigma, \mu, C, b)$ of the stochastic JR-NMM (25) fitted on real EEG data using Algorithm (1) (ii). The red lines correspond to the uniform priors
